# Protein Orientation and Polymer Phase Separation Induced by Poly(methyl methacrylate) Tacticity

**DOI:** 10.1021/acs.langmuir.4c04699

**Published:** 2025-02-03

**Authors:** Natalia Janiszewska, Joanna Raczkowska, Katarzyna Gajos, Kamil Awsiuk

**Affiliations:** † Faculty of Physics, Astronomy and Applied Computer Science, M. Smoluchowski Institute of Physics, Jagiellonian University, Łojasiewicza 11, Kraków 30-348 Poland; ‡ Doctoral School of Exact and Natural Sciences, Jagiellonian University, Łojasiewicza 11, Kraków 30-348, Poland

## Abstract

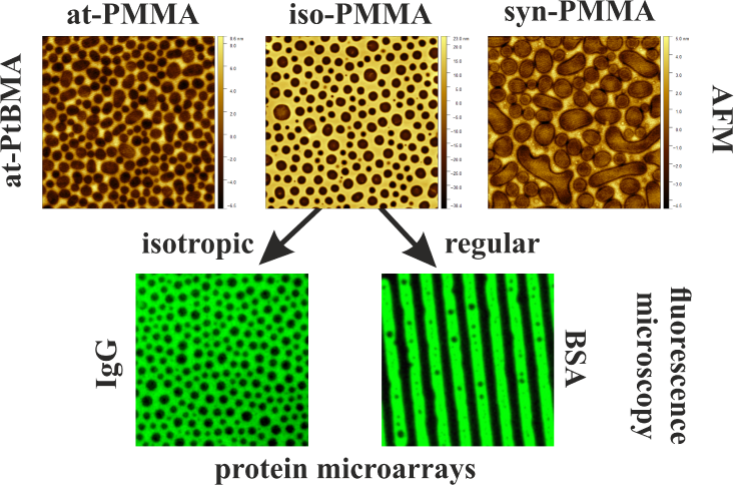

Stereochemistry may affect the physicochemical and biological properties of polymer films that are important for their applications, including substrates for the fabrication of protein microarrays. In this study, we investigated the effect of poly(methyl methacrylate) (PMMA) tacticity on the interaction of polymer thin films with proteins and on the phase separation process in blends with poly(*tert*-butyl methacrylate) (PtBMA). Thin films of isotactic, atactic, and syndiotactic PMMA were studied for topography, surface chemistry, and protein adsorption. Secondary ion mass spectrometry and contact angle measurements revealed a lower surface exposure of polar ester functional groups for iso-PMMA, resulting in the reduced adsorption of albumin and fibrinogen proteins. We also showed that changes in surface chemistry alter the orientation of proteins adsorbed on iso-PMMA through hydrophobic and electrostatic interactions. In addition, blends composed of PMMA and PtBMA, both of different tacticities, were investigated in terms of protein microarray fabrication. The two-dimensional domain structure was obtained by a phase separation process for at-PtBMA blends prepared on silicon substrates modified with amino-silane. Finally, for an isotropic and regular polymer pattern of iso-PMMA/at-PtBMA, the possibility of protein microarray formation on this blend was demonstrated, showing selective adsorption to PtBMA domains and perfect mirroring of the polymer patterns.

## Introduction

In recent decades, polymer-based materials have become one of the basic materials used in medicine and biology, finding numerous applications in diverse fields, including microarrays. Microarrays are ordered collections of different molecules, distributed spatially in a very small space^1,2^ and were originally invented for DNA-based analysis.^3^ However, nowadays, protein microarrays are also used for a variety of purposes in research and clinical studies, mainly for diagnostics and drug development due to their high efficacy in multiplexed detection of biomarkers and antibodies.^4,5^ In response to multiple applications of protein microarrays, different fabrication technologies have been invented to fulfill specific requirements.^6−9^ Among them, protein microarrays based on polymer patterns, formed using soft-lithography, were proposed.^2,10^ Despite the large number of available methods, optimization of protein patterning still requires extensive studies, mainly due to the very complex interaction of proteins with surfaces.^11^ Therefore, for the application of polymer-based materials, it is crucial to determine how they interact with biomolecules, as they can affect the adsorbed molecules differently through their variable properties, such as chemical composition, surface energy, wettability, and roughness. Protein adsorption to surfaces is a common event and is the first step in many biological processes, such as transmembrane signaling or the blood coagulation cascade. It is therefore important to determine how the immobilization of proteins to polymer surfaces occurs.^12^ Immobilization can be defined as the attachment of molecules to a surface, resulting in a reduction or loss of their mobility. In some cases, immobilization can lead to partial or complete loss of protein activity due to random orientation and structural deformation.^13^ To fully maintain biological activity, proteins should be attached to surfaces without affecting their conformation and function. The utilization of functional groups on the surface enables a better interaction between the immobilized protein and the substrate.

Poly(methyl methacrylate) (PMMA) is one of the most widely used polymers in biomedical sciences. The application of PMMA in biomedicine can be divided into four key areas: dentistry, orthopedics, ophthalmology, and device fabrication.^14,15^ PMMA has found its way into dental applications, as its properties, such as ease of processing and pigmenting, good mechanical properties, and above all, its low toxicity, make it the most popular denture base worldwide.^16−18^ Through the use of different polymerization methods, as well as the use of additional substances, it is possible to create a resin tailored to the needs of patients. PMMA resins are trauma-resistant, biocompatible, and resistant to long-term exposure to an aqueous environment. Based on the same properties, PMMA is also used as a main component of artificial bone cements used in orthopedics to provide fixation between bone and implants.^14,19−22^ One of the interesting applications of PMMA is in ophthalmology as a material for foldable intraocular lenses transplanted after cataract surgery.^23^ In addition, thanks to its good physicochemical properties, such as hydrophilicity and easy processing, PMMA is also used in the manufacture of advanced microfluidic cells as well as lab-on-a-chip devices.^24−29^ In addition, chemically modified^30^ or plasma micronanotextured^9^ PMMA surfaces have also been used as polymeric protein and DNA microarrays. Given the wide application of PMMA in medical fields, it is crucial to understand the mechanisms of interaction between the polymer and biomolecules, especially with proteins.

One of the key researchers of PMMA–protein interactions is Takeshi Serizawa, whose research focused on determining the effect of PMMA stereoregularity on the amount of protein adsorbed to the surface. In his work, he focused on studies of the PMMA stereocomplex, a crystalline-like structure with high thermal stability composed of stereoregular isotactic and syndiotactic PMMA chains. He showed that the affinity of the protein for the PMMA stereocomplex was much higher than its affinity for at- or iso-PMMA.^31−33^ Additionally, he demonstrated the ability of the studies to consider the peptide’s recognition of the subtle differences between the thin films of the structure of the stereoregular polymers and the stereocomplex.^33,34^ Despite numerous works on the effect of PMMA stereoregularity on the amount of adsorbed protein, the aspect of changes in protein orientation during the adsorption process has been neglected.

Another interesting polymer is poly(*tert*-butyl methacrylate) (PtBMA). It finds numerous applications in the biomedical field, e.g., as delivery vehicles for hydrophobic chemotherapeutics,^35^ platforms to study the interactions between bacterial cells and polymeric films^36,37^ and materials for protein pattern fabrication.^38^ Our previous studies performed on poly(*tert*-butyl methacrylate) show that polymer stereoregularity strongly modifies the interaction with peptides, proteins, and bacteria.^39^ Differences in group exposure between the isotactic form and atactic and syndiotactic forms affect the orientation and conformation of proteins adsorbed to thin films of these polymers. Exposure of side groups present in the isotactic PtBMA layers causes a change in the orientation of immunoglobulin G (IgG) molecules and affects the conformation of adsorbed bovine serum albumin (BSA).

Motivated by gaps in PMMA research, we conducted a study of the effect of PMMA tacticity on the protein adsorption process. In our study, we used three forms of PMMA stereoregularity: isotactic, atactic, and syndiotactic, to which proteins were adsorbed. PMMA thin films were studied using time-of-flight secondary ion mass spectrometry (ToF-SIMS), atomic force microscopy (AFM), and contact angle measurements (CA) to determine the topography and chemical composition of the surface. Adsorption of proteins was determined using fluorescence microscopy and analyzed semiquantitatively by means of Minkowski measures.^40^ Additionally, changes in protein orientation and conformation were determined by using ToF-SIMS combined with principal component analysis (PCA).

Moreover, we examined the impact of tacticity and substrate on phase separation in thin films of polymer blends composed of PMMA and PtBMA, prepared by two different techniques: spin-casting and h-dipping. The latter enables the fabrication of significantly larger domains with easily tunable spatial dimensions. Then, the ordered polymer patterns were fabricated using a microcontact printing technique. The produced patterns were used to form well-defined arrays of proteins.

## Experimental Section

### Materials

The polymers used in this work: atactic (at-, Mn = 50 × 10^3^, PDI = 1.09; syndio:hetero:isotactic 54:40:6), isotactic (iso-, 52.5 × 10^3^, PDI = 1.16, Iso >98%) and syndiotactic (syn-, Mn = 46 × 10^3^, PDI = 1.8, Syn >79%) poly(methyl methacrylate) (PMMA); atactic (at-, Mn = 523 × 10^3^, PDI = 1.13), isotactic (iso-, Mn = 493 × 10^3^, PDI = 1.27, Iso >85%) and syndiotactic (syn-, Mn = 443 × 10^3^, PDI = 1.1) poly(*tert*-butyl methacrylate) (PtBMA) were purchased from Polymer Source Inc. (Dorval, Montreal, Quebec H9P 2 × 8 Canada). 3-Aminopropyltriethoxysilane (APTES) was obtained from Sigma-Aldrich (Darmstadt, Germany)

Protein adsorption was investigated for bovine serum albumin (BSA, pI = 5.82, mass 69 kDa), goat antimouse IgG (pI ∼ 7, mass 150 kDa), and fibrinogen (pI = 5.8, mass 340 kDa), all labeled with Alexa Fluor 488 and purchased from Invitrogen (USA). Unlabeled bovine serum albumin (BSA; Cohn Fraction V) was obtained from Acros Organics (Thermo Fisher Scientific; Geel, Belgium), and rabbit anti-goat IgG was obtained from Invitrogen (USA). Goat anti-rabbit IgG whole molecule and F(ab)_2_ fragment were obtained from Thermo Fisher, and the Fc fragment of goat IgG was obtained from Rockland Immunochemicals.

### Sample Preparation

Atactic, isotactic, and syndiotactic pure PMMA films were spin-cast on SiO_*x*_ wafers from analytical grade chlorobenzene with a coating speed ω = 2.2 krpm and solution concentration C_P_ = 10 mg/mL. After preparation, all PMMA films were annealed for 1 h at 60 °C. Polymer solutions with a concentration of 20 mg/mL in chlorobenzene were prepared to create isotropic polymer blends of PMMA and PtBMA with different tacticities. The solutions of pure polymers were then mixed together in a 1:1 ratio to form nine PMMA/PtBMA blends. Thin films of the blends were spin-cast (coating speed ω = 2.2 krpm) or were prepared by horizontal-dip (H-dip) coating using the home-built device^41^ on SiO_*x*_ wafers, unmodified and modified with APTES molecules. To fabricate ordered polymer patterns, a microcontact printing method was used to create the regular patterns of APTES on SiO_*x*_ substrates. An APTES solution was applied to the patterned PDMS stamp, and the stamp was then printed onto a silicon substrate. On silicon substrates prepared in this way, films of polymer blends were deposited using the H-dip coating technique with the home-built apparatus.

### Protein Adsorption

Prior to the protein adsorption experiments, all polymer films were annealed for 1 h at 60 °C to remove residual solvent.

Bovine serum albumin (BSA) and fibrinogen (FIB), labeled with Alexa Fluor 488 (λ_abs_ = 496 nm and λ_emit_ = 520 nm), were used to examine the protein adsorption to the PMMA films with different tacticity. Protein solutions (with a concentration of 80 μg/mL for BSA and 30 μg/mL for fibrinogen) were prepared using phosphate saline buffer (PBS, pH = 7.4) (concentration of proteins measured with NanoDrop One (Thermo Scientific)). For the purpose of examining protein adsorption, a drop of protein solution was placed on the polymer film, and the mixture was incubated for a period of 30 min at room temperature. After that, all the samples were rinsed with the buffer and distilled water to remove nonadsorbed proteins and dried under a nitrogen stream.

To verify how polymer tacticity impacts protein orientation, thin PMMA layers on silicon substrates were immersed in a 1 mg/mL polyclonal goat anti-rabbit antibody solution in PBS buffer for 30 min. After the incubation, the samples were carefully washed with buffer and distilled water and dried under a nitrogen stream. Furthermore, F(ab)_2_ and Fc fragments of the used goat anti-rabbit IgG molecules (at a concentration of 500 μg/mL in PBS) were adsorbed on PMMA films for reference purposes.

In order to determine the adsorption of proteins into different components of polymer blends, as well as verify their biological activity, a goat anti-mouse IgG labeled with Alexa Fluor 488 was adsorbed to regular patterns. Prepared samples were immersed in a protein solution at a concentration of 100 μg/mL in PBS buffer. After 30 min, samples were rinsed in buffer and distilled water and dried under a nitrogen stream.

To verify the biological activity of adsorbed antibodies, a binding assay was conducted for isotropic patterns. For the binding assay, samples were incubated with a 100 μg/mL rabbit anti-goat IgG (anti-IgG) solution in 50 mM PBS buffer for 30 min at room temperature. Following washing with buffer, the samples were immersed in blocking buffer (10 mg/mL of BSA solution in 50 mM PBS) for 30 min. Finally, after washing with phosphate buffer and distilled water, the samples were immersed in a 25 μg/mL solution of Alexa Fluor 488-labeled goat anti-mouse IgG (highly cross-adsorbed with rabbit IgG) in blocking buffer for 30 min at room temperature. As the final step, the samples were rinsed in buffer and distilled water and dried under a nitrogen stream.

### Optical Fluorescence Microscopy

To verify protein adsorption to the thin films, the Olympus BX51 optical microscope, equipped with a 100 W halogen lamphouse, camera type DP72, and U-MWIG2 filter (λ_exit_ = 520–550 nm, λ_emit_ > 565 nm), was used. The fluorescence micrographs were recorded for dried samples using the Cell^F program. For each sample, two series of experiments were performed, and for each sample, at least four images were recorded.

### Surface Energy Measurements

The Krüss EasyDrop instrument (DSA15) was used to determine the surface free energy of polymer films. Contact angle measurements were made using the sessile drop technique for formamide and diiodomethane at room temperature. The contact angles were assessed as the average of ten measurements taken at different spots on the same sample surface. Using the Owens– Wendt–Kaelble analytical approach, surface free energy was calculated.

### Atomic Force Microscopy (AFM)

AFM images of polymer films were recorded in noncontact mode (silicon probes with a spring constant of about 2 N/m and resonant frequencies of about 70 kHz) using an Agilent 5500 microscope. AFM images of polymer blends were recorded in contact mode (silicon probes with a spring constant of about 0.2 N/m and resonant frequencies of about 14 kHz) with an Alpha 300R (WITec, Ulm, Germany) microscope. All AFM images were analyzed by using Gwyddion software.

### Profilometry

In order to examine the thickness of the prepared blends, their profiles were recorded by using a Dektak XT (Bruker, Bremen, Germany) profilometer equipped with a 12.5 μm radius stylus. For each sample, a scratch was made on the surface to expose the underlying silicon layer, followed by 4 profiles taken using the standard hill and valley module over the scratch, allowing the thickness of the layer to be determined.

### Time-of-Flight Secondary Ion Mass Spectrometry (ToF-SIMS)

The PMMA surfaces were analyzed prior to and after protein adsorption with a TOF.SIMS 5 (ION-TOF GmbH) instrument. The instrument was equipped with a 30 keV bismuth liquid metal ion gun, and Bi_3_^+^ clusters were used to collect surface spectra from at least four different nonoverlapping spots (200 μm × 200 μm) with a high mass resolution of *m*/Δ*m* > 8000 at C_4_H_5_^+^ (*m*/*z* = 53).

To investigate differences in the structure of polymer films composed of PMMA with different stereoregularity and to detect possible differences in the orientation and conformation of protein molecules adsorbed on iso-, syn-, and at-PMMA films, Principal Component Analysis (PCA) was performed. Prior to PCA, intensities of selected peaks from positive spectra were normalized to the sum of their intensities and mean-centered. PCA was performed using the PLS Toolbox (Eigenvector Research, Manson, WA) for MATLAB (MathWorks, Inc., Natick, MA).

### Confocal Raman Spectroscopy

Confocal Raman spectroscopy measurements were conducted using a Confocal Raman Microscope System Alpha 300R (WITec, Ulm, Germany) with a UHTS300 spectrometer and a DR316B_LD CCD detector with a 600 g/mm grating equipped with a 532 nm laser set and laser power adjusted to 10 mW in front of the objective. Spectral images were acquired using a 50× objective (NA 0.8) EC Epiplan-Neofluar, Zeiss, Germany in the range of 140–3400 cm^–1^ with an integration time of 1 s and a resolution 100 × 100 points per 20 μm × 20 μm. All acquired Raman images were further processed by using WITec ProjectSIX 6.1 software. Images were treated with cosmic ray removal, and a background subtraction procedure was applied using the’shape’ function. After that, spectral images were analyzed using True Component Analysis (TCA), which allowed the identification of individual components that characterize the sample by defining similar spectra as the same component and identifying all pixels in an intensity-distributed image that shared these spectral characteristics. The average spectra of the components were then extracted to allow the molecular information on each component to be identified.

## Results and Discussion

### PMMA Thin Films Characterization

**Figure 1:**
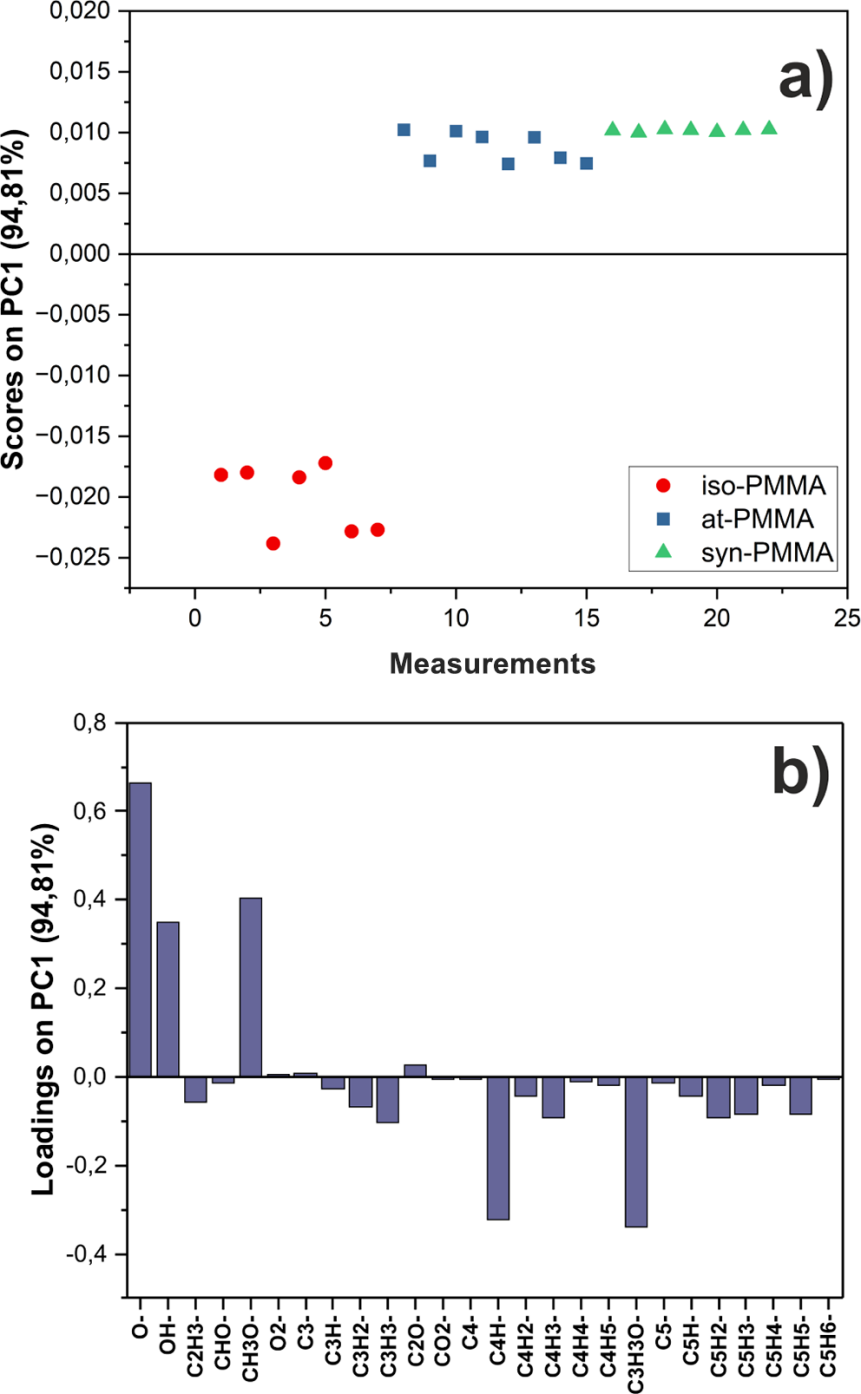
(a) PC1 score plot and (b) corresponding loading plot from principal component analysis of the negative ToF-SIMS signals collected from isotactic (red circles), atactic (blue squares), and syndiotactic (green triangles) PMMA thin films.

Polymer–protein interactions strongly depend on the properties of the polymer film; therefore, we analyzed the polymer film with different techniques prior to protein adsorption. First, the structure of the thin films of all PMMA tacticities was characterized with AFM and profilometry (Figure S1). The thickness of the examined samples was approximately 27 nm, and AFM revealed flat surfaces with RMS below 0.5 nm, and no differences were found between different tacticities. Next, the surface chemistry of polymer films was analyzed with ToF-SIMS combined with multivariate PCA analysis. ToF-SIMS gives information on the uppermost region of the polymer film (1–1.5 nm) so it can be used to evaluate which fragments of the polymer chain are exposed toward the air.^39,42,43^ The data set used for PCA was formed by the intensities of 26 peaks (specified in Table S1) corresponding to negative ion fragments of PMMA polymers. The main direction of uncorrelated major variations, the first principal component (PC1), captures 94.81% of the total variance in the data set (Figure 1a). The PC1 scores plot clearly separates iso-PMMA films having negative PC1 values from at- and syn-PMMA films that exhibit positive PC1 scores (Figure 1a). As can be seen in the loadings plot (Figure 1b), the negative scores are related to the secondary ions C_*x*_H_*y*_^–^ which are alkyl fragments of the main chain (Figure 1b). In turn, positive PC1 scores are due to positive PC1 loadings of oxygen-containing ions (O^–^, OH^–^, CH_3_O^–^, and C_2_O^–^) that are fragments of ester methyl groups of the side chains. These results from PCA of negative ToF-SIMS spectra show that the iso-PMMA surface is preferentially covered with nonpolar groups and contains fewer ester methyl groups at the air side than syn- or at-PMMA films.

Finally, to verify the orientation of polymer side chains shown with ToF-SIMS, the contact angle measurements of sessile droplets for formamide and diiodomethane were done, and the surface free energy (SFE), as well as its polar and dispersive components, were calculated using the Owens–Wendt–Kaelble analytical approach (Table 1).^44^

**Table 1:** Surface Free Energy and its Polar and Disperse Components of iso-, at-, and syn-PMMA

	Dispersive part [mN/m]	Dispersive part [mN/m]	Surface free energy [mN/m]
**iso-PMMA**	39.3 (1.4)	4.3 (0.1)	43.6 (1.5)
**at-PMMA**	39.7 (0.5)	5.3 (0.1)	45.0 (0.6)
**syn-PMMA**	38.5 (2.6)	6.6 (0.1)	45.1 (2.7)

The results, as presented in Table 1, show that the calculated SFE and their dispersive parts, are comparable for all polymers within the framework of estimated uncertainties. In turn, differences are observed for the polar part of the SFE component, which is lower for the iso-PMMA films compared to those of the at- and syn-PMMA films. These results correspond to the PCA results and confirm the surface accumulation of polar ester functional groups for the at- and syn-PMMA films, whereas these groups are hidden deeper for the iso-PMMA. Furthermore, they agree with results obtained by Vanden Eynde et al., who showed that isotacticity leads to a decrease in the concentration of the pendant group of PMMA on the surface.^45^ (The calculated ratio between the pendant group and the main chain is shown in Table S2.)

### Protein Adsorption to PMMA Thin Films with Different Tacticities

In the first step, we evaluated the amount of protein adsorbed onto polymer films made of PMMA with different tacticity. To this end, the fluorescently labeled BSA and fibrinogen were adsorbed onto polymer films. Next, fluorescence images were analyzed semiquantitatively by means of Minkowski measures with a procedure described previously.^40^

**Figure 2:**
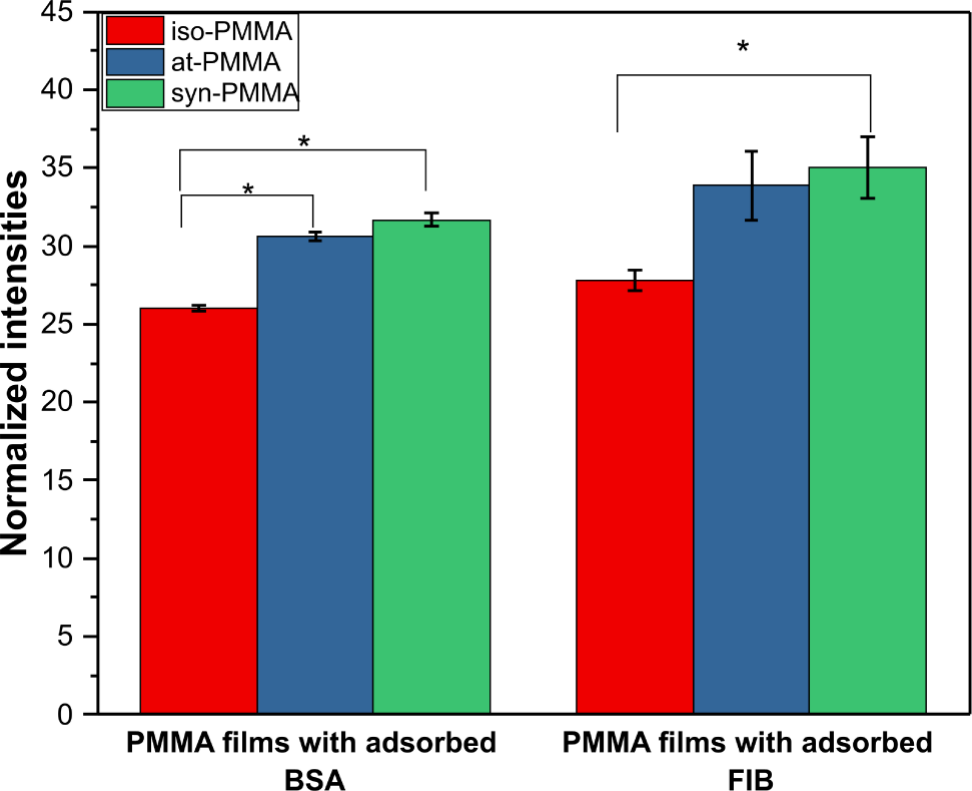
Relative amount of adsorbed bovine serum albumin (BSA) and fibrinogen (FIB) onto thin films composed of iso-, at-, and syn-PMMA.

An examination of the fluorescence micrographs collected for all types of proteins reveals a greater amount of protein molecules adsorbed to syn- and at-PMMA than to iso-PMMA films (Figure 2). This indicates that the tacticity of this polymer affects the amount of adsorbed proteins and is in accordance with the results presented by Serizawa et al.^46−48^

### ToF-SIMS Analysis of Proteins Adsorbed onto PMMA Films

To analyze how polymer tacticity and the differences in film surface chemistry impact the interaction with protein molecules, further studies with ToF-SIMS and PCA were performed for BSA adsorbed onto thin films of PMMA with different tacticities.

**Figure 3:**
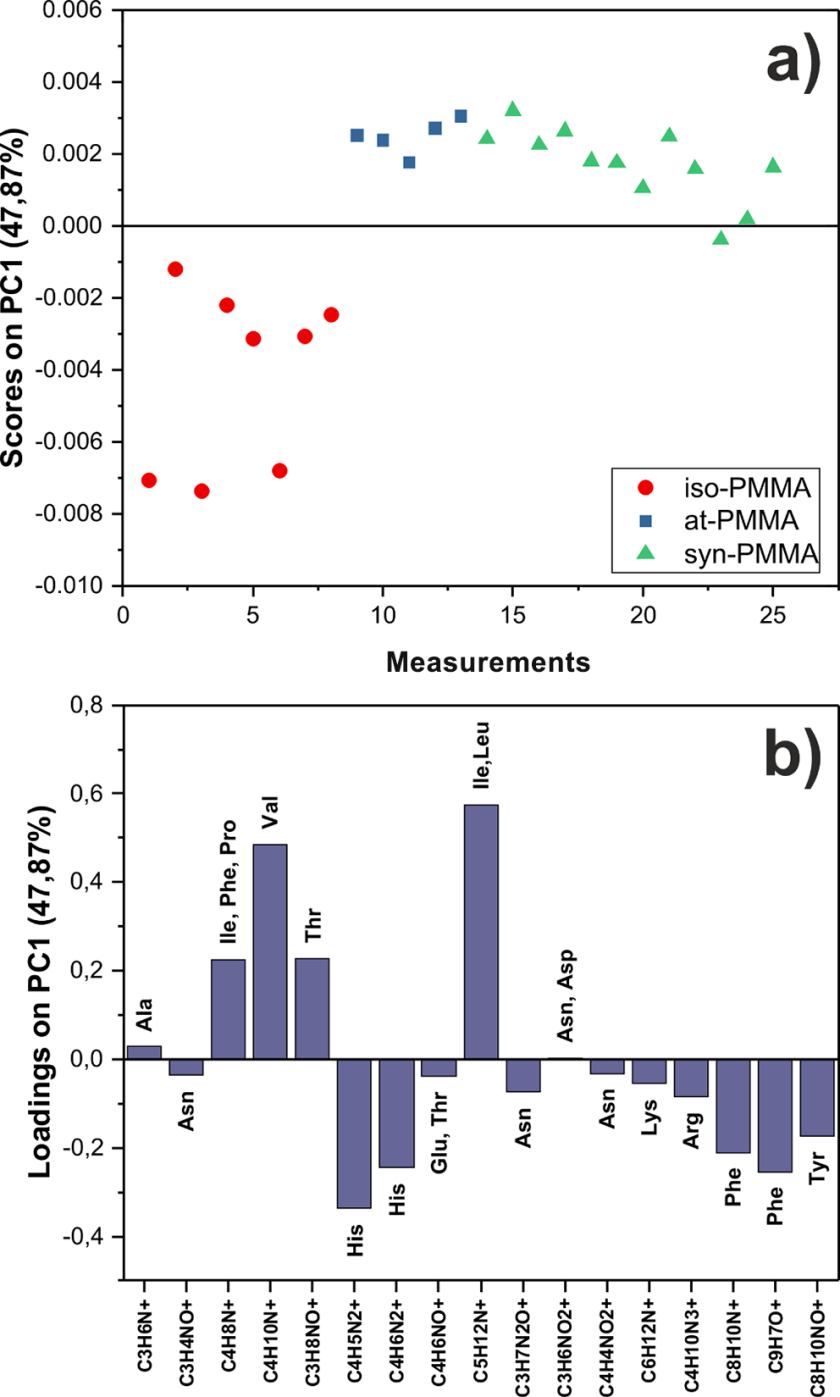
(a) PCA scores plot of the positive ion ToF-SIMS spectra of BSA adsorbed onto iso- (red circle), at- (blue squares), and syn-PMMA (green triangles). (b) Corresponding loadings plot for PC1 with peaks named by origin amino acids.

The result of the principal component analysis performed on a data set containing peaks originating only from amino acids is shown in Figure 3. Each point in the space defined by the scores on the first principal component (PC1) corresponds to a positive ion spectrum in which the peaks listed in Table S3 were included. The PC1 that describes the greatest variation (47.87%) within the data set captures the difference originating from the BSA adsorbed to PMMA with different tacticities. As shown, measured spectra cluster into two groups (Figure 3a). Samples with BSA adsorbed on at- and syn-PMMA are loaded positively in PC1, whereas for iso-PMMA films, they are loaded negatively. Contributions of individual ToF-SIMS peaks to PC1, shown in Figure 3b (called the loadings plot), indicate that the separation comes from signals related to fragments of different amino acids. The negative scores for PC1 are related to fragments of histidine, phenylalanine, and tyrosine. In turn, the positive scores on PC1 are due to positive loadings of ions originating from valine, threonine, isoleucine, and leucine. This implies that regions of BSA rich in those amino acids are exposed out of the PMMA coatings and differ in different tacticities.

One of the reasons might be the denaturation of the BSA molecules. In such a situation, the presence of hydrophobic amino acids, typically hidden deeper in the protein structure, should be observed at the surface of the protein layer. To verify whether the observed changes in the uppermost layer of BSA adsorbed to different PMMA films are related to BSA denaturation, we performed an analysis described in detail in our previous articles.^49,50^ Briefly, in this analysis, a relative measure of the hydrophobicity of the amino acid side chains was defined as the difference in the retention time, Δt_R_, relative to the glycine peptide^51^ and was plotted against the loadings on PC1 (Figure S2). Such a graph presents whether there is any correlation between the hydrophobicity of amino acids and their exposition by protein molecules adsorbed to a specific type of surface (iso-PMMA vs syn- and at-PMMA). The obtained graph clearly shows that no correlation could be indicated, relating PC1 to the conformation changes of BSA molecules adsorbed to PMMA with different tacticities. This suggests that the differences in the composition of the outermost region of the adsorbed BSA layer, revealed by PCA, are related to changes in the BSA molecule orientation. To consider this issue, an analysis of the 3D structure of the BSA molecule was performed.

BSA molecules consist of three domains (Albumin 1, Albumin 2, and Albumin 3) with a comparable number of amino acids (Albumin 1–191 aa, Albumin 2–193 aa, and Albumin 3–198 aa), but with different compositions.^52^ Since the sampling depth of ToF-SIMS is lower than the size of the protein (9 × 5.5 × 5.5 nm^3^), the analysis of amino acids that are exposed in the uppermost region provides information on BSA orientation.^50^

Based on the Protein Data Bank,^53^ the amino acid compositions for all three BSA (4F5S) domains were calculated and combined with PCA results.^52^ Based on this analysis, it can be seen that histidine, phenylalanine, and tyrosine that are exposed by BSA molecules adsorbed to iso-PMMA are more abundant in Albumin 1 than in Albumin 2 and Albumin 3 (His: 8 vs 6 and 3, Phe: 11 vs. 7 and 9, Tyr: 9 vs 7 and 4). In turn, the occurrence of threonine (7 and 7 vs 17) and valine (6 and 11 vs 19) is the highest for Albumin 3. Therefore, the combination of PCA data with BSA structure analysis suggests that BSA adsorbed onto iso-PMMA exposed Albumin 1, whereas Albumin 3 is exposed by protein molecules adsorbed to at- and syn-PMMA films.

**Figure 4:**
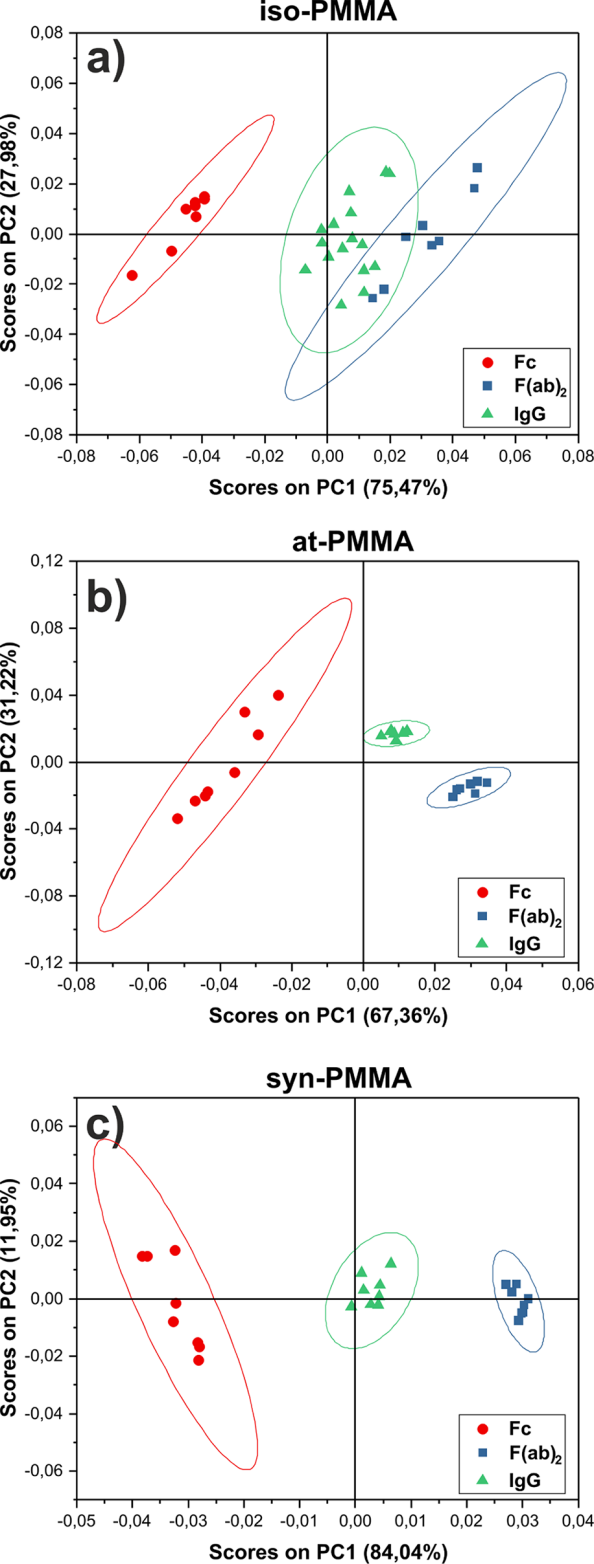
Principal component analysis of whole antibody and their F(ab)_2_ and Fc fragments adsorbed onto (a) iso-PMMA, (b) at-PMMA, and (c) syn-PMMA thin films. Corresponding loading plots are presented in Figure S3.

Since studies conducted for BSA suggest modification of the orientation of protein molecules induced by chemical groups exposed by polymer chains, we did additional studies for IgG. IgG is a Y-shaped molecule for which the orientation of adsorbed protein is extremely important and has a great impact on its activity. Since IgG is a larger and more complex molecule than BSA, to verify the impact of tacticity on the dominant orientation of adsorbed IgG, ToF-SIMS analysis of the whole molecule as well as its fragments (F(ab)_2_ and Fc) adsorbed to at-, iso-, and syn-PMMA was performed. PCA was performed separately for three different tacticities of PMMA, and their score plots are shown in Figure 4. As seen, for each substrate, data points were grouped into three well-separated clusters related to the whole IgG molecule (green) and its F(ab)_2_ (blue) and Fc (red) fragments. For all score plots, PC1 clearly distinguishes between F(ab)_2_ and Fc fragments due to their different composition of amino acids^54^ and the data points from whole molecules are located between these two clusters. However, for iso-PMMA surfaces, overlapping of data points originating from whole IgG molecules and F(ab)_2_ fragments is observed (Figure 4 a) which indicates the IgG molecules’ orientation exposing F(ab)_2_ domain. In turn, for the IgG layer onto at- and syn-PMMA, both F(ab)_2_ and Fc domains of immobilized IgG are probed by ToF-SIMS, which may be considered as a mixed tail-on/head-on orientation of molecules.^55^

The different orientations of protein molecules are caused by different exposures of chemical groups of polymer chains at the surface, depending on the PMMA tacticities. According to the literature, the two main driving forces determining protein adsorption and their orientation are electrostatic and hydrophobic interactions. Therefore, to understand the phenomenon that modifies the orientation of adsorbed proteins, we take into account the charge and aliphatic index of different domains of BSA and IgG molecules.

The charge of a whole protein molecule depends on its isoelectric point (IEP). However, the charge distribution within the molecule is not homogeneous because of the different amino acid compositions, and therefore different protein domains have a different net charge. BSA (IEP about 4.7–5) at pH 7 is negatively charged, and the net charges of different domains are different and equal to −7.8, −9, and −1.3 for Albumins 1, 2, and 3, respectively.^56^ On the other hand, IgG (IEP ∼ 7) at pH 7 is neutral, but the difference in isoelectric points of the F(ab)_2_ and Fc fragments is expected for all IgG antibodies. The isoelectric point of the Fc domain is equal to 5.0–6.0, while that of F(ab)_2_ is usually above 6.5.^55^ As a result, at pH 7, the Fc fragment is negatively charged, while the F(ab)_2_ is positively charged, and the entire IgG molecule has a dipole moment pointing from the Fc to the F(ab)_2_ fragment.

The aliphatic index, the relative volume of aliphatic residues in a protein domain, was obtained based on the ProtParam tool from the ExPASy website (http://www.expasy.org).^57^ For BSA, it was calculated using the amino acid sequences of each domain 4F5S. In turn, for IgG, where the exact sequence was unknown, we did the calculation for a few sequences provided for different rabbit IgG fragments available in the Protein Data Bank (F(ab)_2_: 7RA7, 7D9Z, 7MFR, 4HBC; Fc: 2VUO).^53^ All results are presented in Table 2.

**Table 2:** Aliphatic Index Calculated for BSA Domains and Fc and F(ab)_2_ Fragments of IgG Molecule

protein/domain		aliphatic index
**BSA**	Albumin 1	64.4
	Albumin 2	80.0
	Albumin 3	82.6
**IgG**	Fc	70.4
	F(ab)_2_	69.9

Analysis of the BSA structure shows that all BSA domains are negatively charged, but there is a large difference in the aliphatic index. This suggests that hydrophobic interactions are responsible for the changes in the BSA orientation. Looking at the PCA results for BSA, it can be seen that Albumin 3, with the highest aliphatic index value, is oriented toward the iso-PMMA surface, where nonpolar alkyl fragments (e.g., alpha methyl groups) and PMMA backbone groups are exposed. In turn, Albumin 1, with the lowest aliphatic index value, is oriented toward the syn- and at-PMMA surface, where the polar part of the surface energy is the highest. On the other hand, electrostatic interactions must be more dominant in the orientation of IgG molecules since the values of the aliphatic index for Fc and F(ab)_2_ are comparable and equal to ∼70. Therefore, the ester group exposed at the surface of at- and syn-PMMA repulses the negatively charged Fc fragment of the IgG molecules, and as a result, a part of the molecules adapt a head-on orientation. Similar behavior was observed for PtBMA, where an exposition of the ester group was also exhibited to modify the orientation of adsorbed IgG via electrostatic interactions.

### Impact of Polymer Tacticity on Phase Separation of PMMA/PtBMA

In further studies, we focused on the creation of protein microarrays based on PMMA/PtBMA polymer blends and evaluated how different tacticities impact them. Our previous research pointed to a strong impact of PtBMA tacticity on interactions with peptides, proteins, and bacteria, which were significantly modified for isotactic PtBMA compared to syndiotactic and atactic forms.^39^ Furthermore, protein adsorption experiments to PMMA, presented here (Figure 2) show a greater amount of protein molecules adsorbed to syn- and at-PMMA than to iso-PMMA films. Finally, protein adsorption to PtBMA is higher than to PMMA films (Figure S4) as was also presented by Zemła et al.^40^ Taking into account the results of these experiments, we tried to combine them in order to use PMMA/PtBMA polymer blends for biomedical applications, in particular, as platforms for protein microarrays, by controlling the surface patterns formed by the phase separation process. Thus, the main effort of the studies was the formation of polymeric phase domains with different affinities toward proteins, leading to a preferential adsorption of proteins exclusively on the selected blend component, taking into account the stereoregularity of the polymers and their properties.

In the first step, we studied the topography and morphology of thin films of polymer blends composed of PMMA and PtBMA, both with different stereoregularities, prepared on a SiO_*x*_ substrate.

**Figure 5:**
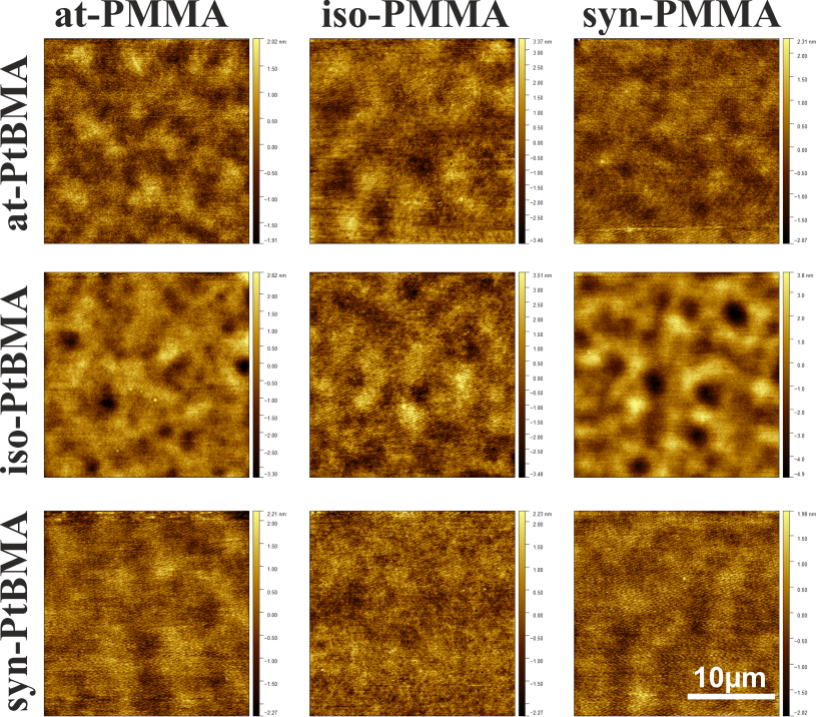
Topography of PtBMA/PMMA polymer blend films spin-cast on SiO_*x*_. The average thickness of the polymer film was 91(2) nm.

**Figure 6:**
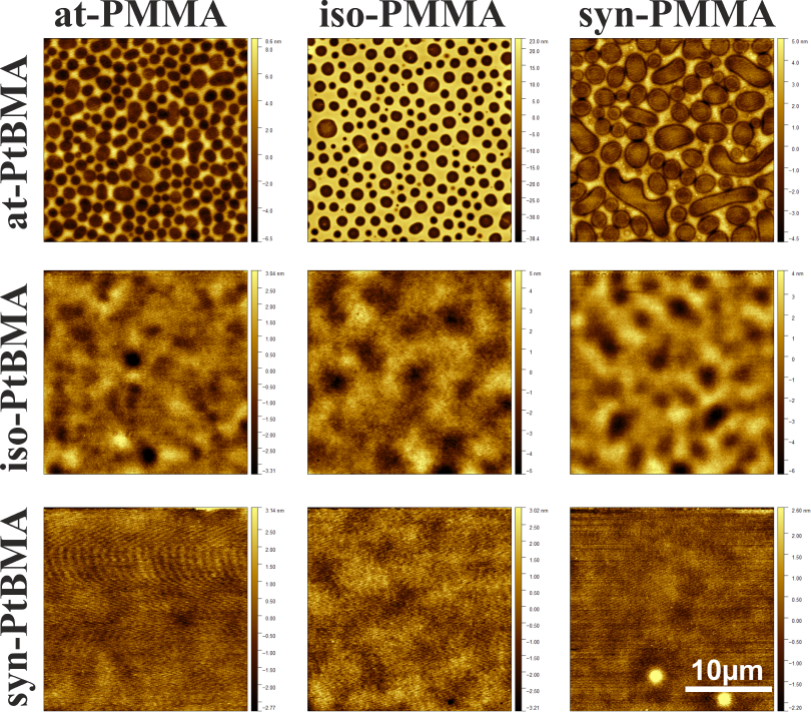
Topography of PtBMA/PMMA polymer blend films spin-cast on APTES. The average thickness of the polymer film was 94(3) nm.

The topography of prepared films (Figure 5), recorded using AFM, reveals completely flat surfaces for all PMMA forms blended with different PtBMA, indicating the formation of a bilayer in this case. Considering the surface energies of both polymers (28.1–29.6 mN/m for PtBMA,^39^ 43.6–45.1 mN/m for PMMA) and the polar SiO_*x*_ substrate (49.4 mN/m), it may be concluded that to minimize the interfacial energy, the bottom layer should be composed of PMMA, with surface energy close to that of the substrate, and the upper layer should be rich in PtBMA, with significantly lower free surface energy. This hypothesis was confirmed by ToF-SIMS measurements, showing the formation of a bilayer structure, with PMMA located on the SiO_*x*_ substrate and covered with an upper PtBMA layer (Figure S5). For all blends composed of at- and iso-PtBMA, the surface undulations are clearly visible. Such undulations may be related to two effects: instabilities of the polymer–polymer interface, caused by van der Waals interactions between the substrate and the upper PtBMA layer, or instabilities of the free surface, resulting from hydrodynamic effects or convection instability during the rapid solvent evaporation.^58^ Observed undulations suggest that the structure of separated phase domain films might be modified by destabilization of the PMMA/PtBMA interface, caused by interactions between the substrate and the upper PtBMA layer, whose strength depends on the interplay between the surface energies of PMMA, PtBMA, and the substrate. Therefore, the SiO_*x*_ surfaces were modified with APTES, characterized by lower surface energy (45.08 mN/m), and used as substrates for spin-casting of thin polymer films composed of PtBMA/PMMA blends. The AFM images recorded for fabricated films confirm a significantly more advanced phase separation process in this case (Figure 6). For blends with syndiotactic PtBMA, which were completely flat on SiO_*x*_, traces of undulations become visible. In turn, for films composed of iso-PtBMA/PMMA, observed wavy structures are significantly better developed compared to SiO_*x*_. Finally, for at-PtBMA mixed with PMMA, phase domains are visible for all blends. It should be noted that the phase separation process that occurs during spin-casting is a very complex process, triggered by decreasing solvent concentration and affected by numerous factors, such as polymer solubility and glass transition temperature, solvent evaporation rate, and specific conditions of film preparation,^59^ as well as the interplay between them. The tacticity of polymers strongly affects all these parameters, and it is not possible to identify a single factor responsible for the final phase domain structure observed in PMMA mixtures with atactic PtBMA.

**Figure 7:**
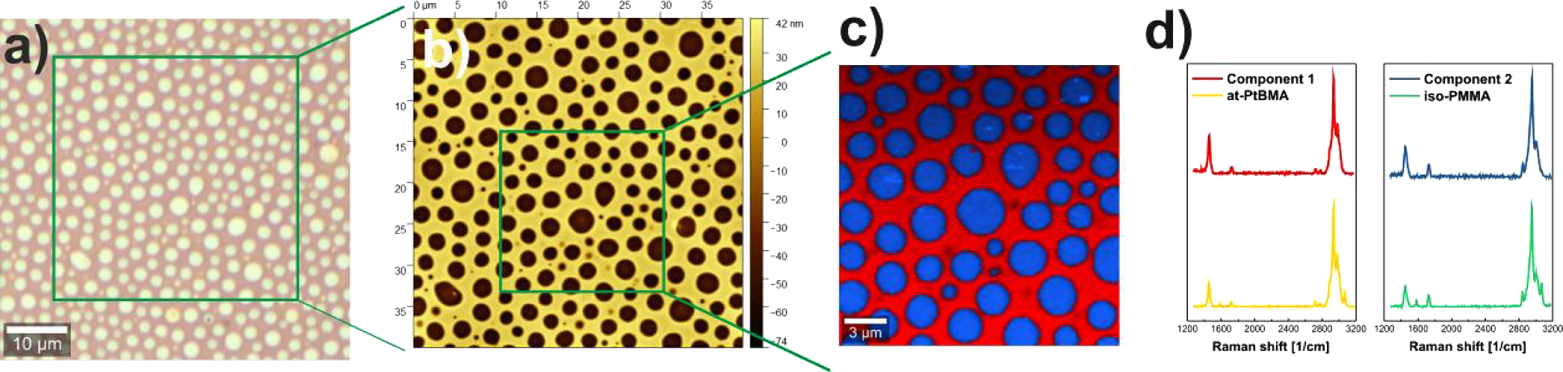
Domain composition in iso-PMMA/at-PtBMA films prepared using h-dipping, determined with (a) optical microscopy, (b) AFM, and (c, d) Raman spectroscopy. (c) The red and blue regions shown in composition maps of TCA of Raman map corresponds to PtBMA and PMMA rich regions, respectively, as it is presented by (d) comparison of Component 1 and Component 2 with reference spectra of pure polymers.

Due to the well-developed phase separation, the preferential orientation of antibodies adsorbed on the at-PtBMA surface (compared to iso-PtBMA) and the lower adsorption of proteins on iso-PMMA (compared to at- and syn-PMMA), the film composed of iso-PMMA and at-PtBMA on an APTES-modified SiO_*x*_ substrate was chosen for further studies. Moreover, to verify in detail domain composition, thin films composed of iso-PMMA and at-PtBMA were examined using Raman spectroscopy. For this purpose, thin films were prepared by using h-dipping, enabling the formation of significantly larger domains than structures accessible by using spin-casting, as determined by using optical microscopy (Figure 7a) and AFM (Figure 7b). The obtained results point to the presence of two components, spatially separated on the sample surface as shown in composition maps of true component analysis (TCA) of the Raman map (Figure 7c) and characterized by different spectra called Component 1 and Component 2 (Figure 7d) corresponding to red and blue areas (Figure 7c), respectively. To verify which components correspond to regions rich in PMMA and PtBMA, their spectra were correlated for the spectral range 2600–3180 cm^–1^, where the most intense signals are observed, with reference spectra measured for pure polymers. The Pearson correlation of Component 1 with reference at-PtBMA and iso-PMMA spectra were 0.99 and 0.85, respectively, whereas for Component 2, correlation with at-PtBMA and iso-PMMA spectra were 0.84 and 0.99, respectively. This shows that the red region is rich in at-PtBMA, whereas the blue one is composed of iso-PMMA.

Comparison of AFM images recorded at the same spot with Raman composition maps clearly reveals that elevated and depressed regions of the sample are formed by different components (Figure 7b,c). To identify them, AFM maps were compared with composition maps determined by Raman spectroscopy, which indicated the formation of iso-PMMA-rich holes in the elevated matrix rich in at-PtBMA.

**Figure 8:**
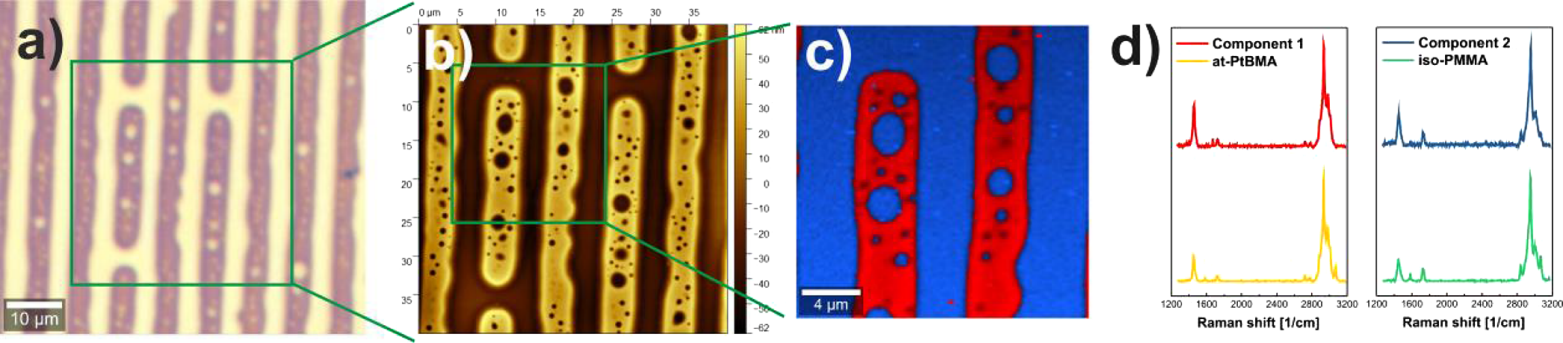
Pattern replication in iso-PMMA/at-PtBMA films prepared using h-dipping, traced using (a) optical microscopy, (b) AFM, and (c, d) Raman spectroscopy. (c) The red and blue regions shown in composition maps of TCA of Raman map corresponds to PtBMA- and PMMA-rich regions, respectively, as it is presented by (d) comparison of Component 1 and Component 2 with reference spectra of pure polymers. Small structures (made from PMMA) observed in the PtBMA-rich domains are due to secondary phase separation.^61^.

For potential applications of examined polymer films as protein microarray templates, polymer domains should be spatially ordered and well-defined. In contrast to isotropic, disordered structures typically formed during phase separation of thin films of polymer mixtures on homogeneous substrates, the presence of a prepatterned substrate leads to the formation of phase domains with long-range order, for appropriately adjusted parameters, such as film thickness, surface and interfacial energies, as well as commensuration between the pattern periodicity λ and the inherent domain scale R.^60^ To produce regular patterns of iso-PMMA/at-PtBMA blends, microcontact printing was applied to premodify the substrate. For this purpose, a poly(dimethylsilane) (PDMS) asymmetric stamp with a stripe-like relief (Figure S6) was used to create a pattern of alternating stripes of SiO_*x*_ and APTES on the substrate (3 μm/5 μm). The AFM analysis of films showed that for a blend composed of iso-PMMA and at-PtBMA spin-cast on homogeneous substrates, the self-organization process leads to the formation of vertical phase domains on APTES (Figure 6) and a bilayer structure on SiO_*x*_ (Figure 5), with PMMA facing the substrate. Thus, for a prepatterned substrate, the SiO_*x*_ stripes should preferentially attract iso-PMMA, and regions covered with APTES, neutral for both system components, should be coated by at-PtBMA, pushed out from SiO_*x*_ regions. Pattern replication driven by preferential interactions of only one blend component with the stripes of one kind was shown to work effectively for the PS:PVP system.^60,61^ This effect was observed also here, and polymer domains formed in films prepared using the h-dipping technique mirrored almost perfectly the pattern imposed by the PDMS stamp, as confirmed by optical microscopy (Figure 8a) and AFM (Figure 8b).

Similarly to isotropic structures, the polymers forming alternating upper and lower regions were identified using Raman spectroscopy and TCA, and again, elevated domains were found to be rich in at-PtBMA (Figure 8c).

### Selective Protein Adsorption to Polymer Patterns

In the final step of our studies, the possibility of protein pattern formation by their selective adsorption to one of the blend components was verified. The analysis of protein adsorption to PMMA with different tacticities showed a similar adsorption rate to at- and syn-PMMA, which was noticeably higher when compared with iso-PMMA. In turn, our previous publication showed that the adsorption of proteins to PtBMA coatings only weakly depends on their stereoregularity and is very effective. Furthermore, protein adsorption to at-PtBMA is more efficient than to iso-PMMA films (Figure S4). Therefore, the blend of weakly adsorbing iso-PMMA and at-PtBMA, capable of high protein adsorption, should enable the formation of protein patterns.

This hypothesis was verified for both types of polymer patterns, i.e., isotropic and regular ones, for two kinds of experiments. For regular patterns (stripes), the selective protein adsorption was verified by immersion of the polymer film in fluorescence-labeled rabbit IgG. After rinsing with PBS and DI water, the sample was dried, and fluorescence images were collected. In turn, for isotropic phase domains, not only was selective adsorption verified but also the biological activity of preabsorbed IgG was checked. This is a crucial and relevant issue for potential biomedical applications of protein patterns, since during adsorption protein molecules can change their orientation and, as a result, block access to specific parts of a protein. To examine whether the selective adsorption to prepatterned polymer surfaces reduces the protein activity, the following experiment was performed. First, the iso-PMMA/at-PtBMA surface patterns were coated with rabbit anti-goat IgG and next incubated in a blocking buffer of BSA, which is commonly used to block any nonspecific interactions. Finally, the samples were incubated in a solution of goat IgG (labeled with Alexa Fluor 488) dissolved in a blocking buffer. In such a procedure, fluorescently labeled goat IgG molecules bind only specifically with preabsorbed anti-IgG; therefore, the fluorescence images correspond to the position of preabsorbed rabbit anti-goat IgG and confirm their biological activity.

**Figure 9:**
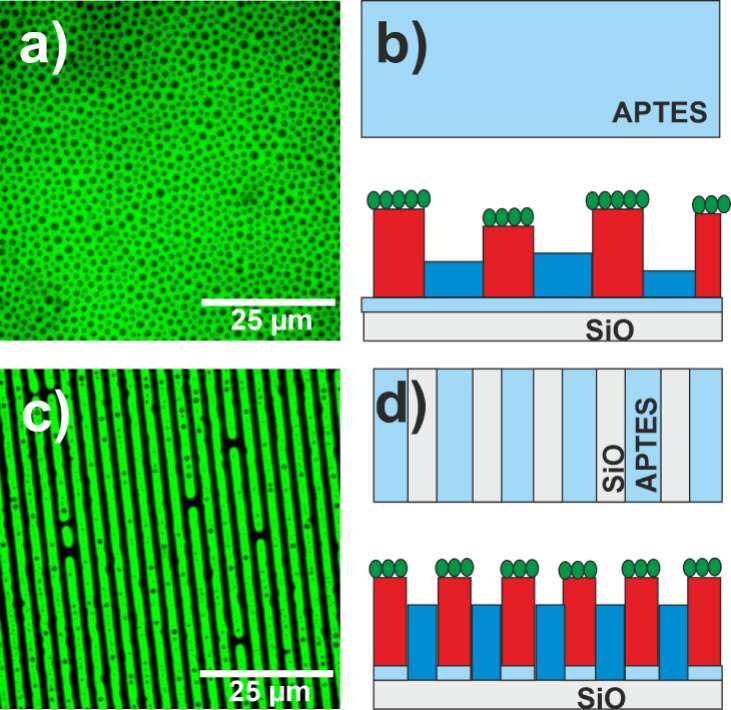
Protein adsorption to isotropic (a-b) and regular (c-d) phase domain patterns formed in iso-PMMA/at-PtBMA films h-dipped on homogeneous APTES (a-b) and patterns of alternating APTES/SiO*_x_* stripes (c-d). Polymer domains with adsorbed protein traced using fluorescence microscopy (a, c) together with schematic illustration of the substrate and proteins adsorbed to the formed PtBMA domains (b, d).

**Figure 10:**
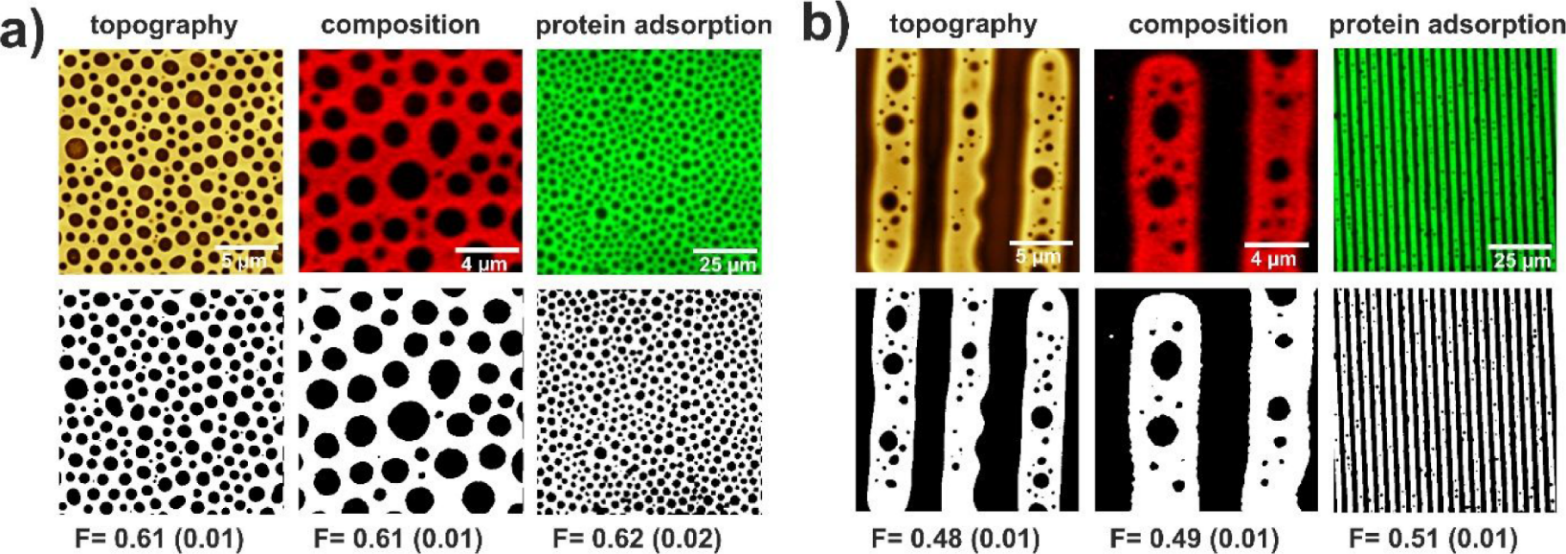
Evaluation of quality of pattern transposition from isotropic (a) and regular (b) polymer patterns into protein arrays based on surface coverage analysis.

Recorded fluorescence micrographs (Figure 9) show the patterns mirroring the phase domains observed on the surface and presented schematically in Figure 9b,d, thus confirming the preferential adsorption of IgG molecules to PtBMA domains and confirming their biological activity. This observation was evaluated qualitatively by comparison of one of the Minkowski functionals, namely surface coverage F.^62^ For this purpose, original AFM topography images, Raman composition maps, and adsorption fluorescence micrographs (upper row in Figure 10) were transformed into black-and-white images using the procedure described in our previous work,^62^ and then the fraction of the white area was calculated, corresponding to elevated regions in AFM images, PtBMA domains in Raman composition maps, and regions with adsorbed protein in fluorescence images. For both isotropic and regular patterns (Figure 10), F showed the same values for all three types of images, confirming the perfect match between the topography and composition of observed domains and indicating the perfect transposition of the polymer pattern into the protein array. This indicates preferential adsorption of proteins to PtBMA-rich phase domains and the retention of biological activity by adsorbed molecules, which is a crucial step in the fabrication of polymeric protein microarrays.

The described blend is an excellent example of the possibility of simplifying and improving the procedure for the production of polymeric protein microarrays by exploiting differences in the stereochemistry of the polymers. By using a suitable polymer mixture, in which the proteins adsorb preferentially to only one of the components (at-PtBMA), and using a stamp of a given design, it is possible to obtain microarrays with high reproducibility.

## Conclusions

In this work, systematic studies were performed, aimed at the investigation of the impact of PMMA tacticity on the properties of thin PMMA films, their interactions with proteins, and the phase separation process in blends with PtBMA.

The thickness and topography of PMMA films, recorded using a profilometer and AFM, respectively, revealed no differences between different tacticities. In turn, the surface chemistry of polymer films analyzed with ToF-SIMS combined with multivariate PCA analysis showed surface accumulation of polar ester functional groups for the at- and syn-PMMA films, whereas these groups are hidden deeper for the iso-PMMA. These results were additionally confirmed by free surface energy calculations, which showed differences for the polar part of the SFE component, lower for iso-PMMA as compared to the at- and syn-PMMA films.

Then, the impact of tacticity on protein adsorption was studied for BSA, IgG, and fibrinogen. The fluorescence micrographs revealed a greater amount of protein molecules adsorbed to syn- and at-PMMA than to iso-PMMA films. In turn, ToF-SIMS analysis of protein orientation performed for BSA and IgG molecules showed different mechanisms of polymer–protein interactions caused by different protein structures and properties. The hydrophobic interactions were found to be responsible for the changes in BSA orientation, whereas electrostatic interactions were more dominant in the orientation of IgG molecules. In the next step, the possibility of fabrication of protein microarrays based on polymer blends was examined using AFM and Raman microscopy for blends composed of PMMA and PtBMA, both of different tacticities. The performed research showed that for SiO_*x*_ substrate phase separation process leads to the formation of a bilayer structure for all PtBMA/PMMA blends, with the lower PMMA layer covered with PtBMA. The two-dimensional domains were observed only for at-PtBMA blended with PMMA with different tacticities when SiO_*x*_ was modified with APTES. This fact was used in the next step, where polymer blends were prepared on a prepatterned substrate with alternating SiO_*x*_ and APTES stripes. In this case, a phase separation process driven by preferential interactions between PMMA and SiO_*x*_ led to the formation of regular polymer patterns. The elevated domains visible in topographical AFM images were composed of PtBMA, whereas the lowered regions were composed of PMMA, as confirmed by Raman microscopy. Then, the possibility of protein pattern formation by their selective adsorption to one of the blend components was verified, showing perfect mirroring of both isotropic and regular polymer patterns by proteins adsorbed to PtBMA domains. Finally, the biological activity of the adsorbed protein was confirmed, pointing to the high potential of the presented materials for application as platforms for protein microarrays.

## Supporting Information Available

AFM images of PMMA films, ToF-SIMS signals selected for Principal Component Analysis, analysis of BSA denaturation, PCA loadings plot of positive ion ToF-SIMS spectra of whole antibody and their fragments, Fluorescence micrographs of IgG and BSA adsorbed onto iso- and at-PtBMA films, ToF-SIMS depth profiling of PtBMA:PMMA blends, images of PDMS stamp (PDF)
Figure S1. Topographic AFM images of thin (a) at-, (b) iso-, and (c) syn-PMMA films with measured thickness; Figure S2. Hydrophobicity of amino acid side chains defined as the difference in retention time (Δt_R_) relative to glycine peptide plotted against the loadings on PC 1 for BSA adsorbed to PMMA thin films; Figure S3. PCA loadings plot of positive ion ToF-SIMS spectra of whole antibody, their F(ab)_2_ and Fc fragments adsorbed into iso-PMMA (a), at-PMMA (b) and syn-PMMA (c); Figure S4. Fluorescence micrographs show higher IgG and BSA adsorption to at-PtBMA than to iso-PMMA films; Figure S5. ToF-SIMS depth profiling of PtBMA:PMMA blends on the SiO_*x*_ substate; Figure S6. Photograph (a) and AFM scan (b) of the asymmetric stamp with the stripe-like relief; Table S1. ToF-SIMS signals and their mases selected for Principal Component Analysis from mass spectra of PMMA films; Table S2. Main chain and ester group parameters for negative ToF-SIMS spectra of PMMA films and their ratios; Table S3. ToF-SIMS signals, their mases and amino acid association selected for Principal Component Analysis from mass spectra of BSA adsorbed on PMMA films (PDF)
