# Supplementary material for: Protein Orientation and Polymer Phase Separation Induced by Poly(methyl methacrylate) Tacticity
Source: Langmuir. 2025 Feb 3;41(5):3549–60. doi: 10.1021/acs.langmuir.4c04699 (PMC11823595; doi:10.1021/acs.langmuir.4c04699)
Supplement: Supplementary file 1 — la4c04699_si_001.pdf [file la4c04699_si_001.pdf]

Protein orientation and polymer phase separation induced by poly(methyl methacrylate) tacticity

Natalia Janiszewska[1,2], Joanna Raczkowska[1], Katarzyna Gajos[1], Kamil Awsiuk[1*]

[1]Jagiellonian University, Faculty of Physics, Astronomy and Applied Computer Science, M. Smoluchowski Institute of Physics, Łojasiewicza 11, 30-348 Kraków, Poland

[2]Jagiellonian University, Doctoral School of Exact and Natural Sciences, Łojasiewicza 11, 30-348 Kraków, Poland

[*] Corresponding author E-mail: kamil.awsiuk@uj.edu.pl

Abstract

Stereochemistry may affect physicochemical and biological properties of polymers films that are important for their applications, including substrates for the fabrication of protein microarrays. In this study, we investigated the effect of poly(methyl methacrylate) (PMMA) tacticity on the interaction of polymer thin films with proteins and on the phase separation process in blends with poly(tert-butyl methacrylate) (PtBMA). Thin films of isotactic, atactic and syndiotactic PMMA were studied for topography, surface chemistry and protein adsorption. Secondary ion mass spectrometry and contact angle measurements revealed a lower surface exposure of polar ester functional groups for iso-PMMA, resulting in reduced adsorption of albumin and fibrinogen proteins. We also showed that changes in surface chemistry alter the orientation of proteins adsorbed on iso-PMMA through hydrophobic and electrostatic interactions. In addition, blends composed of PMMA and PtBMA, both of different tacticities, were investigated in terms of the protein microarray fabrication. The 2-dimensional domain structure was obtained by a phase separation process for at-PtBMA blends prepared on silicon substrates modified with amino-silane. Finally, for an isotropic and regular polymer pattern of iso-PMMA/at-PtBMA, the possibility of protein microarrays formation on this blend was demonstrated, showing selective adsorption to PtBMA domains and perfect mirroring of the polymer patterns.

Graphical abstract

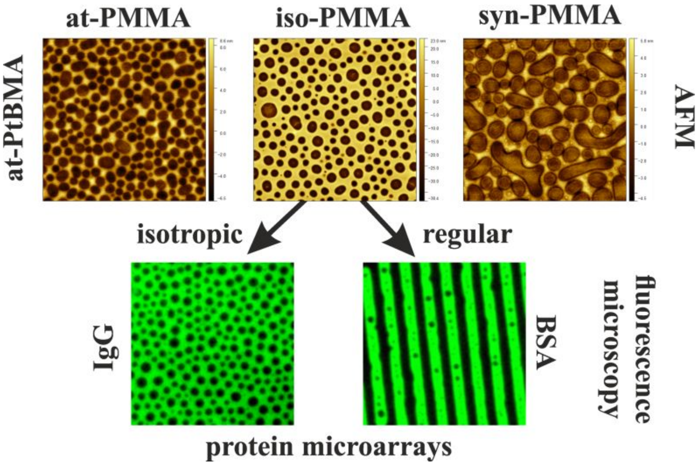

INTRODUCTION

In recent decades, polymer-based materials have become one of the basic materials used in medicine and biology, finding numerous applications in diverse fields, including microarrays. Microarrays are ordered collections of different molecules, distributed spatially in a very small space [1,2] and were originally invented for DNA-based analysis [3]. However, nowadays also protein microarrays are used for a variety of purposes in research and clinical studies, mainly for diagnostics and drug development due to their high efficacy in multiplexed detection of biomarkers and antibodies[4–6]. In response to multiple applications of protein microarrays, different fabrication technologies have been invented to fulfill specific requirements [7–10]. Among them, also protein microarrays based on polymer patterns, formed using soft-lithography were proposed [2,11]. Despite the large number of available methods, optimization of protein patterning still requires extensive studies, mainly due to the very complex interaction of proteins with surfaces [12]. Therefore, for the application of polymer-based materials, it is crucial to determine how they interact with biomolecules, as they can affect the adsorbed molecules differently through their variable properties, such as chemical composition, surface energy and wettability, and roughness. Protein adsorption to surfaces is a common event and is the first step in many biological processes such as the trans membrane signaling or blood coagulation cascade. It is therefore important to determine how the immobilization of proteins to polymer surfaces occurs [13]. Immobilization can be defined as the attachment of molecules to a surface, resulting in a reduction or loss of their mobility. In some cases, immobilization can lead to partial or complete loss of protein activity, due to random orientation and structural deformation [14]. To fully maintain biological activity, proteins should be attached to surfaces without affecting their conformation and function. The utilization of functional groups on the surface enables a better interaction between the immobilized protein and the substrate.

Poly(methyl methacrylate) (PMMA) is a one of the most widely used polymers in biomedical sciences. The application of PMMA in biomedicine can be divided into four key areas: dentistry, orthopedics, ophthalmology and device fabrication [15,16]. PMMA has found its way into dental applications, as their properties such as ease of processing and pigmenting and good mechanical properties and above all its low toxicity make them the most popular denture base worldwide [17–19]. Through the use of different polymerization methods as well as the use of additional substances, it is possible to create a resin tailored to the needs of patients. PMMA resins are trauma-resistant, biocompatible and resistant to long-term exposure to an

aqueous environment. Based on the same properties, PMMA is also used as a main component of artificial bone cements used in orthopedics to provide a fixation between bone and implants [15,20–23]. One of the interesting applications of the PMMA is in the ophthalmology as material for foldable intraocular lenses transplanted after the cataract surgery [24]. In addition, thanks to its good physico-chemical properties such as hydrophilicity and easy processing, PMMA is also used in the manufacture of advanced microfluidic cells as well as lab-on-a-chip devices [25–30]. In addition, chemically modified [31] or plasma micronanotextured [10] PMMA surfaces have also been used as polymeric protein and DNA microarrays. Given the wide application of PMMA in medical fields, it is crucial to understand the mechanisms of interaction between the polymer and biomolecules, especially with proteins.

One of the key researchers of PMMA-protein interactions is Takeshi Serizawa, whose research focused on determining the effect of PMMA stereoregularity on the amount of protein adsorbed to the surface. In his work, he put the impact on studies of the PMMA stereocomplex, a crystalline-like structure with a high thermal stability composed of stereoregular isotactic and syndiotactic PMMA chains. He showed that the affinity of the protein for the PMMA stereocomplex was much higher than its affinity for at- or iso- PMMA [32–34]. Additionally, he performed the ability of the studies considered the peptide to recognize the subtle differences between the thin films of structure of the stereoregular polymers and the stereocomplex [34,35]. Despite numerous works on the effect of PMMA stereoregularity on the amount of adsorbed protein, the aspect of changes in protein orientation during the adsorption process has been neglected.

Another interesting polymer is poly(tert-butyl methacrylate) (PtBMA). It finds numerous applications in biomedical field, e.g. as delivery vehicles for hydrophobic chemotherapeutics [36], platforms to study the interactions between bacterial cells and polymeric films [37,38] and materials for protein pattern fabrication [39]. Our previous studies performed for poly(tert-butyl methacrylate) present that polymer stereoregularity strongly modified the interaction with peptides, proteins and bacteria [40]. Differences in group exposure between the isotactic form, and atactic and syndiotactic forms affected the orientation and conformation of proteins adsorbed to thin films of these polymers. Exposure of side groups present for the isotactic PtBMA layers caused a change in the orientation of immunoglobulin G (IgG) molecules and affected the conformation of adsorbed bovine serum albumin (BSA).

Motivated by gaps in PMMA research, we conducted a study of the effect of PMMA tacticity on protein adsorption process. In our study, we used three forms of PMMA stereoregularity isotactic, atactic and syndiotactic to which proteins were adsorbed. PMMA thin

films were studied using time-of-flight second ions mass spectrometry (ToF-SIMS), atomic force microscopy (AFM) and contact angle measurements (CA) to determine the topography and chemical composition of the surface. Adsorption of proteins was determined using fluorescence microscopy and analyzed semi-quantitatively by means of Minkowski measures [41]. Additionally, changes in protein orientation and conformation were determined using ToF-SIMS combined with principal component analysis (PCA).

Moreover, we examined impact of tacticity and substrate on phase separation in thin films of polymer blends composed of PMMA and PtBMA, prepared by two different techniques: spin-casting and h-dipping. The latter one enables fabrication of significantly larger domains, with easily tunable spatial dimensions. Then, the ordered polymer patterns were fabricated using microcontact printing technique. Produced patterns were used to form well-defined arrays of proteins.

EXPERIMENTAL SECTION

Materials

The polymers used in this work: atactic (at-, Mn= 50 x 10^3, PDI= 1.09; syndio:hetero:isotactic 54:40:6), isotactic (iso-, 52.5 x 10^3, PDI= 1.16, Iso > 98%) and syndiotactic (syn-, Mn= 46 x 10^3, PDI= 1.8, Syn>79%) poly (methyl methacrylate) (PMMA); atactic (at-, Mn= 523 x 10^3, PDI= 1.13) isotactic (iso-, Mn= 493 x 10^3, PDI= 1.27, Iso > 85%) and syndiotactic (syn-, Mn= 443 x 10^3, PDI=1.1) poly(tert-butyl methacrylate) (PtBMA) were purchased from Polymer Source Inc. (Dorval (Montreal) Quebec H9P 2x8 Canada). 3-aminopropyltriethoxysilane (APTES) was obtained from Sigma-Aldrich (Darmstadt, Germany)

Protein adsorption was investigated for bovine serum albumin (BSA, pI=5.82, mass 69 kDa), goat anti-mouse IgG (pI~7, mass 150 kDa) and fibrinogen (pI=5.8, mass 340 kDa) all labeled with Alexa Fluor 488 and purchased from Invitrogen (USA). Unlabeled bovine serum albumin (BSA; Cohn Fraction V) was obtained from Acros Organics (Thermo Fisher Scientific; Geel, Belgium) and rabbit anti-goat IgG was obtained from Invitrogen (USA), goat anti-rabbit IgG whole molecule and F(ab)2 fragment from Thermofisher and Fc fragment of goat IgG from Rockland Immunochemicals.

Sample preparation

Atactic, isotactic and syndiotactic pure PMMA films were spin-cast on SiO_x wafers from analytical grade chlorobenzene with coating speed ω = 2.2 krpm and solution

concentration C_P = 10 mg/ml. After preparation, all PMMA films were annealed for 1 h at 60 °C. Polymer solutions with a concentration of 20mg/ml in chlorobenzene were prepared to create isotropic polymer blends of PMMA and PtBMA with different tacticities. The solutions of pure polymers were then mixed together in a 1:1 ratio to form nine PMMA/PtBMA blends. Thin films of the blends were spin-cast (coating speed ω = 2.2 krpm) or were prepared by horizontal-dip (H-dip) coating using the home-built device [42] on SiO_x wafers unmodified and modified with APTES molecules. To fabricate ordered polymer patterns micro-contact printing method was used to create the regular patterns of APTES on SiO_x substrates. An APTES solution was applied to the patterned PDMS stamp and the stamp was then printed onto a silicon substrate. On silicon substrates prepared in this way, films of polymer blends were deposited using the H-dip coating technique using the home-built apparatus.

Protein adsorption

Prior to the protein adsorption experiments, all polymer films were annealed for 1 h at 60 °C to remove residual solvent.

Bovine serum albumin (BSA), and fibrinogen (FIB) all labeled with Alexa Fluor 488 (λ_{abs} = 496 nm and λ_{emit} = 520 nm) were used to examine the protein adsorption to the PMMA films with different tacticity. Protein solutions (with a concentration 80 μg/ml for BSA, and 30μg/ml for fibrinogen), were prepared using phosphate saline buffer (PBS, pH = 7.4) (concentration of proteins measured with NanoDrop One (Thermo Scientific)). For the purpose of examination of protein adsorption, a drop of protein solution was placed on the polymer film and incubated for a period of 30 min at room temperature. After that, all the samples were rinsed with the buffer and distilled water to remove non-adsorbed proteins and dried under nitrogen stream.

To verify how polymer tacticity impact on protein orientation thin PMMA layers on silicon substrates were immersed in 1 mg/ml polyclonal goat anti-rabbit antibody solution in PBS buffer for 30 minutes. After the incubation, the samples were carefully washed with buffer, distilled water and dried under a nitrogen stream. Furthermore, $F(ab)_2$ and Fc fragments of the used goat anti-rabbit IgG molecules (at a concentration of 500 μg/ml in PBS) were adsorbed on PMMA films for reference purposes.

In order to determine the adsorption of proteins into different components of polymer blends as well as verification of their biological activity, a goat anti-mouse IgG labeled with Alexa Fluor 488 was adsorbed to regular patterns. Prepared samples were immersed in protein

solution in the concentration of 100 μg/mL in PBS buffer. After 30 minutes samples were rinsed in buffer and distilled water and dried under a nitrogen stream.

To verified biological activity of adsorbed antibodies a binding assay was conducted for isotropic patterns. For binding assay samples were incubated with a 100 μg/mL rabbit anti-goat IgG (anti-IgG) solution in 50 mM PBS buffer for 30 minutes at room temperature. Following washing with buffer, the samples were immersed in blocking buffer (10 mg/ml BSA solution in 50 mM PBS) for 30 min. Finally, after washing with phosphate buffer and distilled water, the samples were immersed in a 25μg/mL solution of Alexa Fluor 488-labelled goat anti-mouse IgG (highly cross-adsorbed with rabbit IgG) in blocking buffer for 30 min at room temperature. As the final step the samples were rinsed in buffer and distilled water and dried under a nitrogen stream.

Optical Fluorescence Microscopy

To verified protein adsorption to the thin films the Olympus BX51 optical microscope, equipped with a 100 W halogen lamphouse, camera type DP72 and U-MWIG2 filter (λ_{exit} = 520-550 nm, λ_{emit}>565 nm) was used. The fluorescence micrographs were recorded for dried samples using the Cell^F program. For each sample two series of experiments were performed and for each sample at least four images were recorded.

Surface Energy Measurements

The Kruss EasyDrop instrument (DSA15) was used to determine the surface free energy of polymer films. Contact angles measurements were made using the sessile drop technique for formamide and diiodomethane at room temperature. The contact angles were assessed as the average of ten measurements taken at different spots on the same sample surface. Using the Owens - Wendt – Kaelble analytical approach, surface free energy was calculated.

Atomic Force Microscopy (AFM)

AFM images of polymer films were recorded in non-contact mode (silicon probes spring constant about 2 N/m and resonant frequencies about 70 kHz) using an Agilent 5500 microscope working. AFM images of polymer blends were recorded in contact mode (silicon probes spring constant about 0.2 N/m and resonant frequencies about 14 kHz) with an Alpha 300R (WITec, Ulm, Germany) microscope. All AFM images were analyzed using Gwyddion software.

Profilometry

In order to examine the thickness of the prepared blends, their profiles were recorded using a Dektak XT (Bruker, Bremen, Germany) profilometer equipped with a 12.5 μm radius stylus. For each sample, a scratch was made on the surface to expose the underlying silicon layer, followed by 4 profiles taken using the standard hill and valley module over the scratch, allowing the thickness of the layer to be determined.

Time-of-Flight Secondary Ion Mass Spectrometry (ToF-SIMS)

The PMMA surfaces were analyzed prior to and after proteins adsorption with a TOF.SIMS 5 (ION-TOF GmbH) instrument. The instrument was equipped with 30 keV bismuth liquid metal ion gun and Bi_3^+ clusters were used to collect surface spectra from at least four different non-overlapping spots (200 μm × 200 μm) with a high mass resolution of m/Δm > 8000 at C_4H_5+ (m/z = 53).

To investigate differences in the structure of polymer films composed of PMMA with different stereoregularity and to detect possible differences in the orientation and conformation of protein molecules adsorbed on iso-, syn- and at-PMMA films, Principal Component Analysis (PCA) was performed. Prior to PCA, intensities of selected peaks from positive spectra were normalized to sum of their intensities and mean centered. PCA was performed using PLS Toolbox (Eigenvector Research, Manson, WA) for MAT-LAB (MathWorks, Inc., Natick, MA).

Confocal Raman spectroscopy

Confocal Raman spectroscopy measurements were conducted using a Confocal Raman Microscope System Alpha 300R (WITec, Ulm, Germany) with a UHTS300 spectrometer and a DR316B_LD CCD detector with 600 g/mm grating equipped with a 532 nm laser set and laser power adjusted to 10 mW in front of the objective. Spectral images were acquired using a 50× objective ((NA 0.8) EC Epiplan-Neofluar, Zeiss, Germany) in the range of 140–3400 cm^{-1} with an integration time of 1 second and a resolution 100×100 points per 20 μm × 20 μm. All acquired Raman images were further processed using the WITec ProjectSIX 6.1 software. Images were treated with a cosmic ray removal and a background subtraction procedure was applied using the 'shape' function. After that spectra images were analyzed using True Component Analysis (TCA), which allowed the identification of individual components that characterize the sample by defining similar spectra as the same component and identifying all pixels in an intensity-distributed image that shared these spectral characteristics. The average spectra of the components were then extracted to allow the molecular information of each component to be identified.

RESULTS and DISCUSSION

PMMA thin films characterization

Polymer-protein interactions strongly depend on the properties of the polymer film therefore, we analyzed the polymer film with different techniques prior to protein adsorption. First, the structure of the thin films of all PMMA tacticities was characterized with AFM and profilometry (Fig. S1 in Supporting Information). The thickness of the examined samples was approximately 27 nm and AFM revealed flat surfaces with RMS below 0.5 nm, and no differences were found between different tacticities. Next, the surface chemistry of polymers films was analyzed with ToF-SIMS combined with multivariate PCA analysis. ToF-SIMS gives information on the uppermost region of the polymer film (1–1.5 nm) so it can be used to evaluate which fragments of the polymer chain are exposed toward the air.[40,43,44] The data set used for PCA was formed by the intensities of 26 peaks (specified in Table S1) corresponding to negative ion fragments of PMMA polymers. The main direction of uncorrelated major variations, the first principal component (PC1), captures 94.81% of the total variance in the data set (Fig. 1a). The PC1 scores plot clearly separates iso-PMMA films, having negative PC1 values, from at- and syn-PMMA films that exhibit positive PC1 scores (Fig. 1a). As can be seen at Loadings plot (Fig. 1b), the negative scores are related to the secondary ions $C_xH_y^-$ which are alkyl fragments of the main chain (Fig. 1b). In turn, positive PC1 scores are due to positive PC1 loadings of oxygen containing ions (O^-, OH^-, CH_3O^-, and C_2O^-) that are fragments of ester methyl groups of the side chains. These results from PCA of negative ToF-SIMS spectra show that the iso-PMMA surface is preferentially covered with nonpolar groups and contains fewer ester methyl groups at the air side than syn- or at-PMMA films.

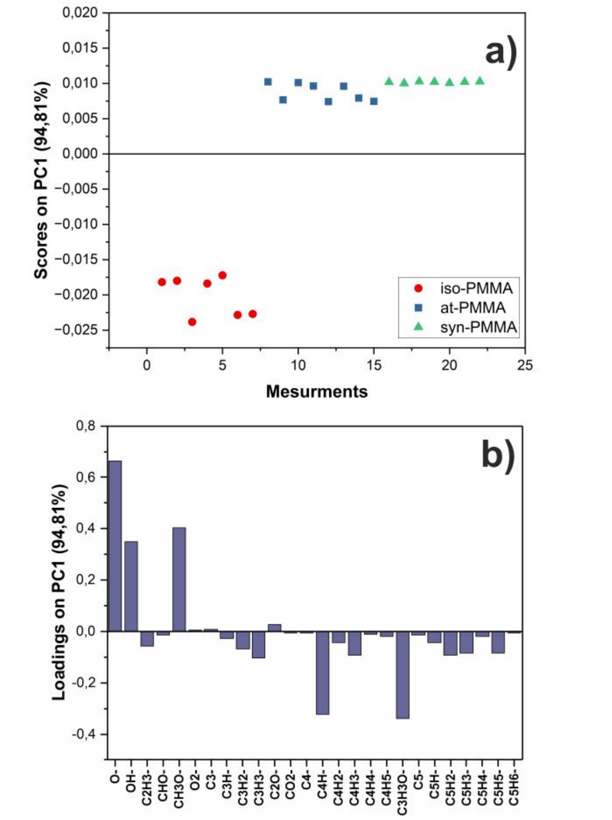

Figure 1. (a) PC1 scores plot and (b) corresponding loadings plot from principal component analysis of the negative ToF-SIMS signals collected from isotactic (red circles), atactic (blue squares), and syndiotactic (green triangles) PMMA thin films.

Finally, to verify the orientation of polymer side chains shown with ToF-SIMS the contact angles measurement of sessile droplets for formamide and diiodomethane was done and the surface free energy (SFE) as well as its polar and dispersive components were calculated using Owens - Wendt – Kaelble analytical approach (Table. 1) [45].

Table 1. Surface free energy and its polar and disperse components of iso-, at-, and syn-PMMA.

	**Dispersive part [mN/m]**	**Polar part [mN/m]**	**Surface free energy [mN/m]**
**iso-PMMA**	39.3 (1.4)	4.3 (0.1)	43.6 (1.5)
**at-PMMA**	39.7 (0.5)	5.3 (0.1)	45.0 (0.6)
**syn-PMMA**	38.5 (2.6)	6.6 (0.1)	45.1 (2.7)

The results, as presented in Table 1, show that the calculated SFE as well as their dispersive parts are comparable for all polymers in the frame of estimated uncertainties. In turn, the differences are observed for the polar part of the SFE component, which is lower for the iso-PMMA films compared to those of the at- and syn-PMMA films. These results correspond to the PCA results and confirms the surface accumulation of polar ester functional groups for the at- and syn-PMMA films, whereas these groups are hidden deeper for the iso-PMMA. Furthermore, they agree with results obtained by X. Vanden Eynde et al., who showed that isotacticity leads to a decrease in the concentration of the pendant group of PMMA on the surface.[46] (The calculated ratio between the pendant group and the main chain is shown in Table S2.)

Protein adsorption to PMMA thin films with different tacticity

In the first step we have evaluated the amount of protein adsorbed into polymer films made of PMMA with different tacticity. To this end the fluorescently labeled BSA and fibrinogen were adsorbed to polymer films. Next fluorescence images were analyzed semi-quantitatively by means of Minkowski measures with procedure described previously [41].

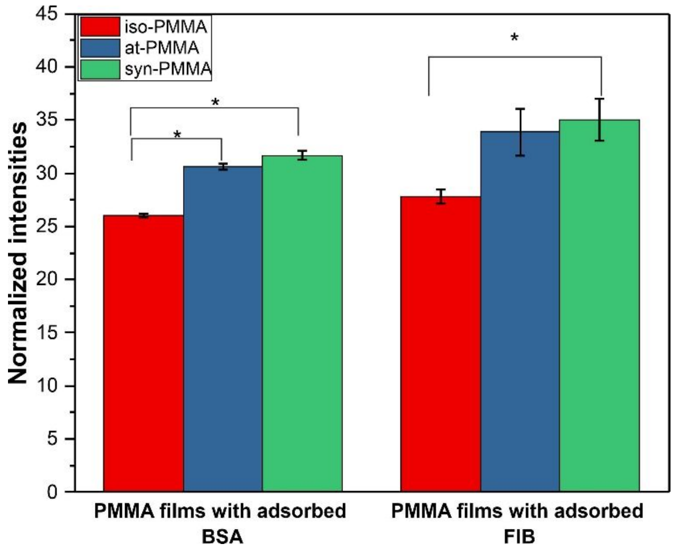

Figure 2. Relative amount of adsorbed bovine serum albumin (BSA) and fibrinogen (FIB) onto thin films composed of iso-, at-, and syn- PMMA.

An examination of the fluorescence micrographs collected for all types of proteins reveals a greater amount of protein molecules adsorbed to syn- and at-PMMA than to iso-PMMA films (Fig. 2). This indicates that the tacticity of this polymer affects the amount of adsorbed proteins and is in accordance with the results presented by Serizawa et al. [47–49].

ToF-SIMS analysis of proteins adsorbed to PMMA films

To analyze how polymer tacticity and the differences in film surface chemistry impact the interaction with proteins molecules, further studies with ToF-SIMS and PCA were performed for BSA adsorbed to thin films of PMMA with different tacticities.

The result of the principal component analysis performed on a data set containing peaks originating only from amino acids is shown in Fig. 3. Each point in the space defined by the scores on the first principal components (PC1) corresponds to a positive ion spectrum in which the peaks listed in Table S3 in the Supporting Information were included. The PC1 that describes the greatest variation (47.87%) within the data set captures the difference originating from the BSA adsorbed to PMMA with different tacticities. As shown, measured spectra cluster into two groups (Fig. 3a). Samples with BSA adsorbed on at- and syn-PMMA are loaded positively in PC1, whereas for iso-PMMA films they are loaded negatively. Contributions of individual ToF-SIMS peaks to PC1, shown in Fig. 3b (called loadings plot), indicate that the separation comes from signals related to fragments of different amino acids. The negative scores for PC1 are related to fragments of histidine, phenylalanine, and tyrosine. In turn, the positive scores on PC1 are due to positive loadings of ions originating from valine, threonine, isoleucine, and leucine. This implies that regions of BSA rich in those amino acids are exposed out of the PMMA coatings and differ for different tacticities.

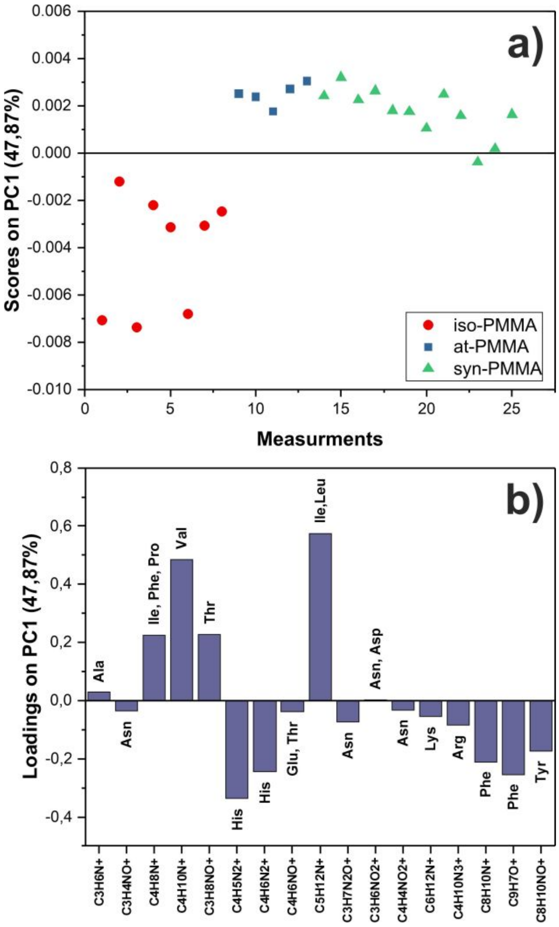

Figure 3. (a) PCA scores plot of the positive ion ToF-SIMS spectra of BSA adsorbed to iso- (red circle), at- (blue squares) and syn-PMMA (green triangles). (b) Corresponding loadings plot for PC1 with peaks named by origin amino acids.

One of the reasons might be the denaturation of BSA molecules. In such a situation, the presence of hydrophobic amino acids typically hidden deeper in the protein structure should be observed at the surface of the protein layer. To verify whether the observed changes in the uppermost layer of BSA adsorbed to different PMMA films are related with BSA denaturation, we performed an analysis described in details in our previous articles [50][51]. Briefly, in this analysis, a relative measure of the hydrophobicity of the amino acid side chains was defined as the difference in the retention time, Δt_R, relative to glycine peptide [52] and was plotted against the loadings on PC1 (Fig. S2 in the Supporting Information). Such a graph presents whether there is any correlation between the hydrophobicity of amino acids and their exposition by protein molecules adsorbed to a specific type of surface (iso-PMMA vs. syn- and at-PMMA). The obtained graph clearly shows that no correlation could be indicated, relating PC1 with the conformation changes of BSA molecules adsorbed to PMMA with different tacticities. This suggests that the differences in the composition of the outermost region of adsorbed BSA layer,

revealed by PCA, are related to changes in the BSA molecule orientation. To consider this issue an analysis of the 3D structure of the BSA molecule was performed.

BSA molecules consists of three domains (Albumin 1, Albumin 2, and Albumin 3) with a comparable number of amino acids (Albumin 1 − 191 aa, Albumin 2 − 193 aa, and Albumin 3 − 198 aa), but their different compositions[53]. Since the sampling depth of ToF-SIMS is lower than the size of the protein ($9 \times 5.5 \times 5.5$ nm^3), the analysis of amino acids that are exposed in the upper most region provides information of BSA orientation.[51]

Based on the Protein Data Bank [54], the amino acid compositions for all three BSA (4F5S) domains were calculated and combined with PCA results.[53] Based on this analysis, it can be seen that histidine, phenylalanine, and tyrosine that are exposed by BSA molecules adsorbed to iso-PMMA are more abundant in Albumin 1 than in Albumin 2 and Albumin 3 (His: 8 vs 6 and 3, Phe: 11 vs 7 and 9, Tyr: 9 vs 7 and 4). In turn, the occurrence of threonine (7 and 7 vs 17) and valine (6 and 11 vs 19) is the highest for Albumin 3. Therefore, the combination of PCA data with BSA structure analysis suggests that BSA adsorbed onto iso-PMMA exposed Albumin 1, whereas, Albumin 3 is exposed by protein molecules adsorbed to at- and syn-PMMA films.

Since studies conducted for BSA suggest modification of the orientation of protein molecules induced by chemical groups exposed by polymer chains, we did and additional studies for IgG. IgG is a Y-shaped molecule for which the orientation of adsorbed protein is extremely important and has a great impact on their activity. Since IgG is a larger and more complex molecule than BSA, to verify the impact of tacticity on the dominant orientation of adsorbed IgG ToF-SIMS analysis of whole molecule as well as its fragments ($F(ab)_2$ and Fc) adsorbed to at-, iso-, and syn-PMMA was performed. PCA was performed separately for three different tacticities of PMMA and their score plots are shown at Fig. 4. As seen, for each substrate, data points were grouped into three, well separated clusters related to the whole IgG molecule (green) and their $F(ab)_2$ (blue) and Fc (red) fragments. For all score plots PC1 clearly distinguishes between $F(ab)_2$ and Fc fragments due to their different composition of amino acids [55] and the data points from whole molecules are located between these two clusters. However, for iso-PMMA surfaces overlapping of data points originating from whole IgG molecules and $F(ab)_2$ fragments is observed (Fig. 4 a) that indicates the IgG molecules orientation exposing $F(ab)_2$ domain. In turn, for the IgG layer onto at- and syn-PMMA both $F(ab)_2$ and Fc domains of immobilized IgG are probed by ToF-SIMS, that may be considered as mixed tail-on/head-on orientation of molecules. [56]

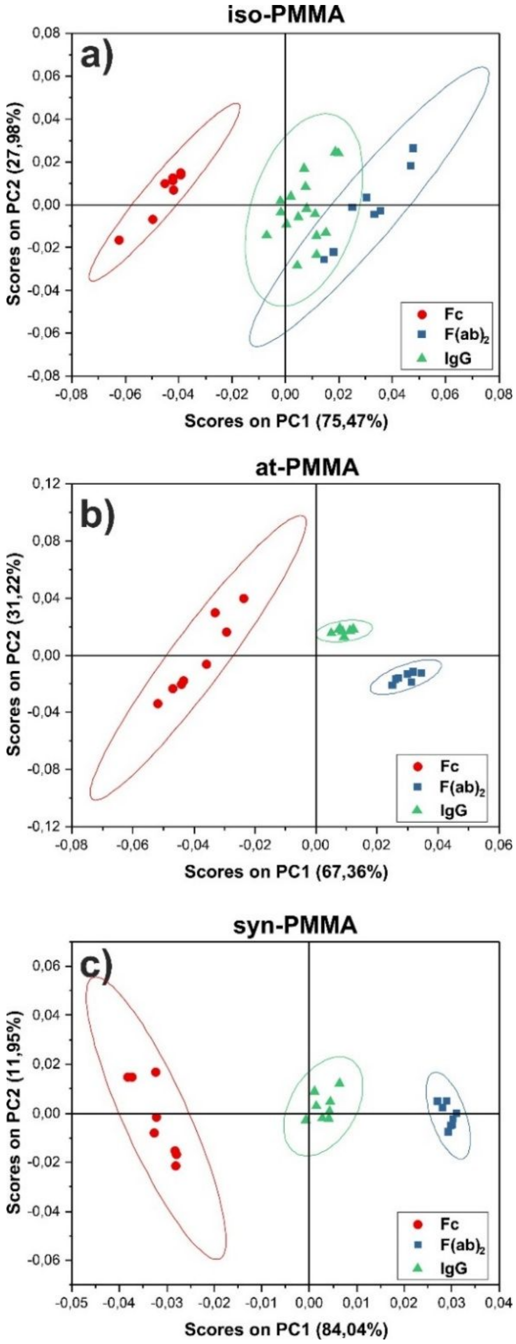

Figure 4. Principal component analysis of whole antibody and their F(ab)$_2$ *and Fc fragments adsorbed to a) iso-PMMA, b) at-PMMA and c) syn-PMMA thin films. Corresponding loading plots are presented in Supporting Information (Fig. S3).*

The different orientations of protein molecules are caused by different expositions of chemical groups of polymer chains at the surface, depending on the PMMA tacticities. According to the literature, the two main driving forces determining protein adsorption and their orientation are electrostatic and hydrophobic interactions. Therefore, to understand the phenomenon that modifies orientation of adsorbed proteins, we take into account charge and aliphatic index of different domains of BSA and IgG molecules.

The charge of a whole protein molecule depends on its isoelectric point (IEP). However, the charge distribution within the molecule is not homogeneous, because of the different amino acid compositions, and therefore different protein domains have a different net charge. BSA (IEP about 4.7−5) at pH 7 is negatively charged and the net charges of different domains are different and equal to −7.8, −9, and −1.3 for Albumins 1, 2, and 3, respectively.[57] On the other hand, IgG (IEP ~ 7) at pH 7 is neutral, but the difference in an isoelectric points of the $F(ab)_2$ and Fc fragments is expected for all IgG antibodies. Isoelectric point of Fc domain is equal to 5.0–6.0, while of $F(ab)_2$ it is usually above 6.5. [56] As a result, at pH 7, the Fc fragment is negatively charged, while the $F(ab)_2$ is positively charged and the entire IgG molecule has a dipole moment pointing from an Fc to an $F(ab)_2$ fragment.

The aliphatic index, the relative volume of aliphatic residues in a protein domain, was obtained based on the ProtParam tool from the ExPASy website (http://www.expasy.org) [58]. For BSA it was calculated using the amino acid sequences of each domain 4F5S. In turn, for IgG where the exact sequence was unknown, we did the calculation for a few sequences provided for different rabbit IgG fragments available in Protein Data Bank ($F(ab)_2$: 7RA7, 7D9Z, 7MFR, 4HBC; Fc: 2VUO) [54]. All results are presented in Table 2.

Table 2. Aliphatic index calculated for BSA domains and Fc and $F(ab)_2$ fragments of IgG molecule

**protein/domain**		**aliphatic index**
**BSA**	Albumin 1	64.4
	Albumin 2	80.0
	Albumin 3	82.6
**IgG**	Fc	70.4
	$F(ab)_2$	69.9

Analysis of the BSA structure shows that all BSA domains are negatively charged but there is a large difference in the aliphatic index. This suggests that hydrophobic interactions are responsible for the changes in BSA orientation. Looking at the PCA results for BSA, it can be seen that Albumin 3 with the highest aliphatic index value is oriented toward the iso-PMMA surface where nonpolar alkyl fragments (e.g., alpha methyl groups) and PMMA back bone groups are exposed. In turn, Albumin 1 with the lowest aliphatic index value is oriented towards the syn- and at-PMMA surface, where the polar part of the surface energy is the highest. On the

other hand, electrostatic interactions must be more dominant in the orientation of IgG molecules since the values of the aliphatic index for Fc and $F(ab)_2$ are comparable and equal ~70. Therefore, the ester group exposed at the surface of at- and syn-PMMA repulses the negatively charged Fc fragment of the IgG molecules, and as a result, a part of molecules adapt head-on orientation. Similar behavior was observed for PtBMA, where an exposition of the ester group was also exhibited to modify the orientation of adsorbed IgG via electrostatic interactions.

Impact of polymer tacticity on phase separation of PMMA/PtBMA

In the further studies we focused on creation of proteins microarrays based on PMMA/PtBMA polymer blends and evaluation how different tacticity impact them. Our previous research pointed to strong impact of PtBMA tacticity on interactions with peptides, proteins, and bacteria, which were significantly modified for isotactic PtBMA as compared to syndiotactic and atactic form [40]. Furthermore, protein adsorption experiments to PMMA, presented here (Fig. 2) show greater amount of protein molecules adsorbed to syn- and at-PMMA than to iso-PMMA films. Finally, protein adsorption to PtBMA is higher than to PMMA films (Fig. S4) as it was also presented by J. Zemla et al. [41]. Taking into account the results of these experiments, we tried to combine them in order to use PMMA/PtBMA polymer blends for biomedical applications, in particular as platforms for protein microarrays, by controlling the surface patterns formed by the phase separation process. Thus, the main effort of the studies was the formation of polymeric phase domains with different affinity towards proteins, leading to a preferential adsorption of proteins exclusively on the selected blend component, taking into account the stereoregularity of the polymers and their properties.

In a first step, we studied the topography and morphology of thin films of polymer blends composed of PMMA and PtBMA, both with different stereoregularity, prepared on SiO_x substrate.

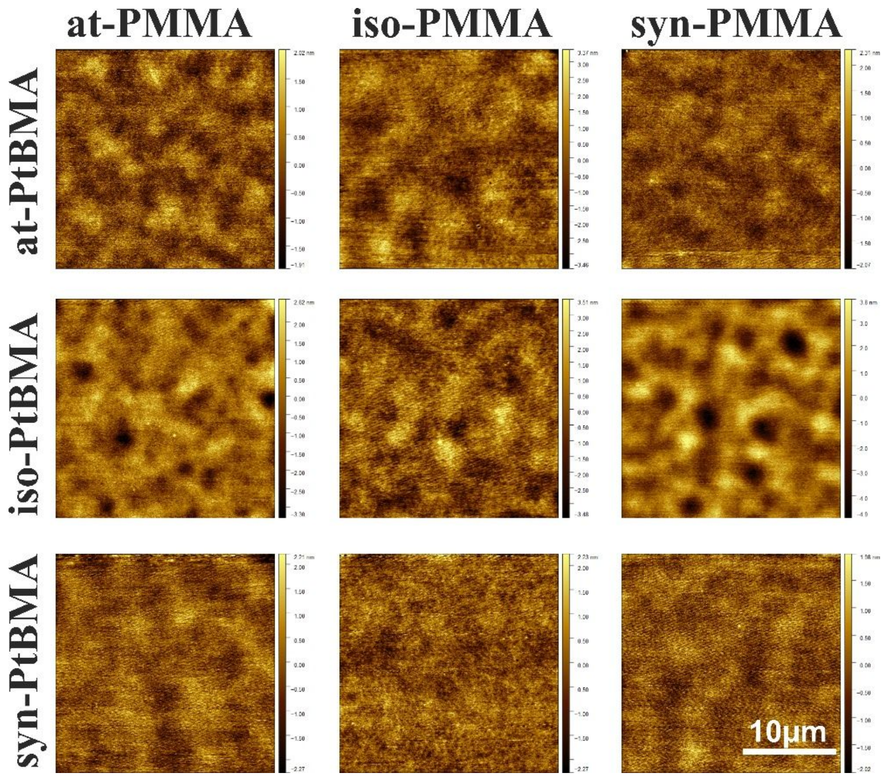

Figure 5. Topography of PtBMA/PMMA polymer blend films spin-cast on SiO_x. The average thickness of polymer film was 91(2) nm.

The topography of prepared films (Fig. 5), recorded using AFM, reveals completely flat surfaces for all PMMA forms blended with different PtBMA indicating formation of bilayer in this case. Considering the surface energies of both polymers (28.1-29.6 mN/m for PtBMA,[40] 43.6-45.1 mN/m for PMMA) and polar SiO_x substrate (49.4 mN/m), it may be concluded, that to minimize the interfacial energy, the bottom layer should be composed of PMMA, with surface energy close to the one of the substrate, and the upper layer should be rich in PtBMA, with significantly lower free surface energy. This hypothesis was confirmed by ToF-SIMS measurements, showing the formation of bilayer structure, with PMMA located on the SiO_x substrate and covered with upper PtBMA layer (Fig. S5). For all blends composed with at- and iso-PtBMA the surface undulation are clearly visible. Such undulations may be related with two effects, instabilities of polymer-polymer interface, caused by van der Waals interactions between substrate and the upper PtBMA layer or instabilities of free surface, resulting from hydrodynamic effect or convection instability during the rapid solvent evaporation [59]. Observed undulations suggest that the structure of separated phase domains films might be modified by destabilization of PMMA/PtBMA interface, caused by interactions between substrate and upper PtBMA layer, which strength depends on the interplay between surface energies of PMMA, PtBMA and substrate. Therefore, the SiO_x surfaces were modified with

APTES, characterized by lower surface energy (45.08 mN/m) and used as substrates for spin-casting of thin polymer films composed of PtBMA/PMMA blends. The AFM images recorded for fabricated films confirm significantly more advanced phase separation process in this case (Fig. 6). For blends with syndiotactic PtBMA, which were completely flat on SiO_x, traces of undulations become visible. In turn, for films composed of iso-PtBMA/PMMA observed wavy structures are significantly better developed, compared to SiO_x. Finally, for at- PtBMA mixed with PMMA, phase domains are visible for all blends. It should be noted, that phase separation process that occurs during spin-casting is a very complex process, triggered by decreasing solvent concentration and affected by numerous factors, such as polymer solubility and glass transition temperature, solvent evaporation rate and specific conditions of film preparation [60] as well as the interplay between them. The tacticity of polymers, strongly affects all these parameters, and it is not possible to point to the single one responsible for the final phase domain structure, observed for PMMA mixtures with atactic PtBMA form.

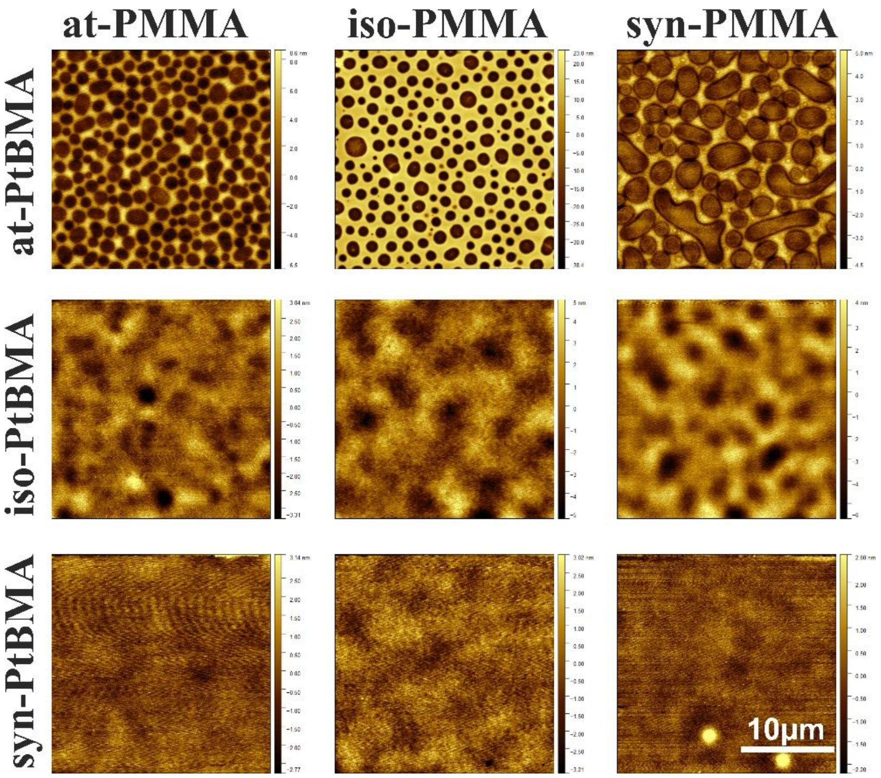

Figure 6. Topography of PtBMA/PMMA polymer blend films spin-cast on APTES. The average thickness of polymer film was 94(3) nm.

Due to the well-developed phase separation, the preferential orientation of antibodies adsorbed on the at-PtBMA surface (compared to iso-PtBMA) and the lower adsorption of proteins on iso-PMMA (compared to at- and syn-PMMA), the film composed of iso-PMMA

and at-PtBMA on an APTES-modified SiO_x substrate was chosen for further studies. Moreover, to verify in detail domain composition, thin films composed of iso-PMMA and at- PtBMA were examined using Raman spectroscopy. For this purpose, thin films were prepared using h-dipping, enabling formation of significantly larger domains than structures accessible using spin-casting, as determined using optical microscopy (Fig. 7a) and AFM (Fig. 7b). Obtained results point to the presence of two components, spatially separated on the sample surface as shown in composition maps of true component analysis (TCA) of Raman map (Fig. 7c) and characterized by different spectra called Component 1 and Component 2 (Fig. 7d) corresponding to red and blue areas (Fig. 7c), respectively. To verify which components correspond to regions rich in PMMA and PtBMA their spectra were correlated for spectral range 2600-3180 cm^{-1}, where the most intense signals are observed, with reference spectra measured for pure polymers. The Pearson correlation of Component 1 with reference at-PtBMA and iso-PMMA spectra were 0.99 and 0.85, respectively, whereas for Component 2 correlation with at-PtBMA and iso-PMMA spectra were 0.84 and 0.99, respectively. These shows that red region are rich in at-PtBMA, whereas blue one are composed of iso-PMMA.

Comparison of AFM images recorded at the same spot with Raman composition maps clearly reveals that elevated and depressed regions of the sample are formed by different components (Fig 7 b and c). To identify them, AFM maps were compared with composition maps determined by Raman spectroscopy and indicated formation of iso-PMMA-rich holes in the elevated matrix rich in at-PtBMA.

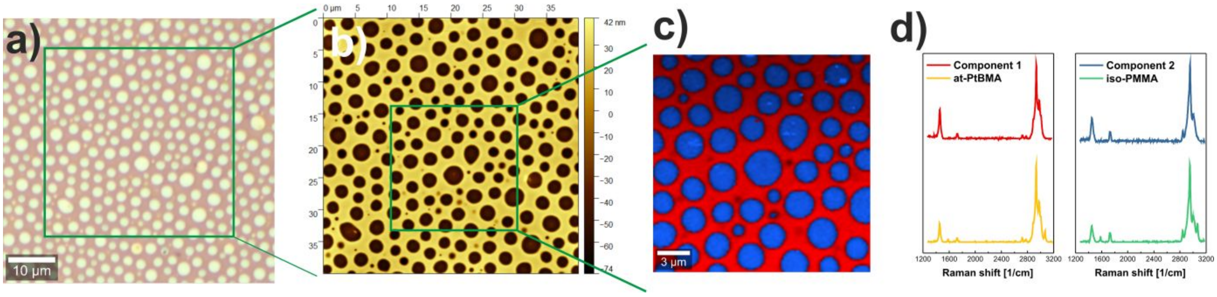

Figure 7. Domain composition in iso-PMMA / at- PtBMA films prepared using h-dipping, determined with (a) optical microscopy, (b) AFM and (c, d) Raman spectroscopy. (c) The red and blue regions shown in composition maps of TCA of Raman map corresponds to PtBMA and PMMA rich regions, respectively, as it is presented by (d) comparison of Component 1 and Component 2 with reference spectra of pure polymers.

For potential application of examined polymer films as protein microarray templates, polymer domains should be spatially ordered and well defined. In contrast to isotropic, disordered structures formed typically during phase separation of thin films of polymer

mixtures on homogeneous substrates, the presence of a pre-patterned substrate leads to the formation of phase domains with long-range order, for appropriately adjusted parameters, such as film thickness, surface and interfacial energies as well as commensuration between the pattern periodicity λ and the inherent domain scale R [61]. To produce regular patterns of iso-PMMA/at-PtBMA blends, microcontact printing was applied to pre-modify substrate. For this purpose poly(dimethylsilane) (PDMS) asymmetric stamp with the stripe-like relief (Fig. S6) was used to create pattern of alternating stripes of SiO_x and APTES on the substrate (3µm / 5 µm). The AFM analysis of films showed, that for a blend composed of iso-PMMA and at-PtBMA spin cast on homogeneous substrates, self-organization process leads to formation of vertical phase domains on APTES (Fig. 6) and bilayer structure on SiO_x (Fig. 5), with PMMA facing the substrate. Thus, for pre-patterned substrate the SiOx stripes should preferentially attract iso-PMMA, and regions covered with APTES and neutral for both system components should be coated by at-PtBMA, pushed out from SiO_x regions. Pattern replication driven by preferential interactions of only one blend component with the stripes of one kind was shown to work effectively for PS:PVP system.[61,62] This effect was observed also here, and polymer domains formed in films prepared using h-dipping technique mirrored almost perfectly the pattern imposed by PDMS stamp as confirmed by optical microscopy (Fig. 8a) and AFM (Fig 8b).

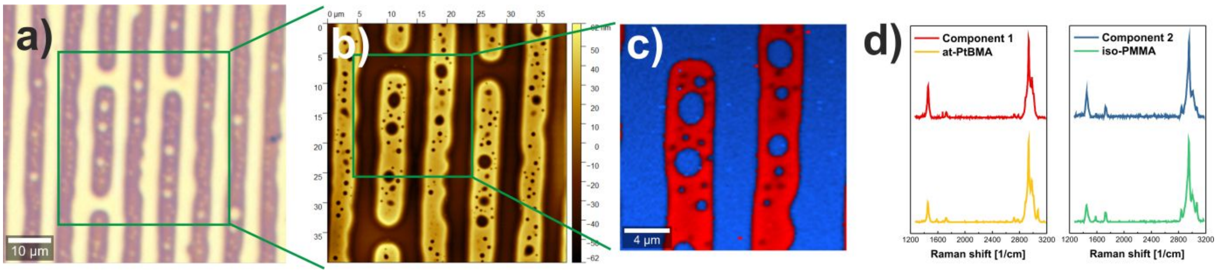

Figure 8. Pattern replication in iso-PMMA / at- PtBMA films prepared using h-dipping, traced using (a) optical microscopy, (b) AFM and (c, d) Raman spectroscopy. (c) The red and blue regions shown in composition maps of TCA of Raman map corresponds to PtBMA and PMMA rich regions, respectively, as it is presented by (d) comparison of Component 1 and Component 2 with reference spectra of pure polymers. Small structures (made from PMMA) observed in the PtBMA-rich domains are due to secondary phase separation. [62]

Similarly as for isotropic structures, also here the polymers forming alternating upper and lower regions were identified using Raman spectroscopy and TCA, and again elevated domains were found to be rich in at-PtBMA (Fig. 8c).

Selective Protein Adsorption to Polymer Patterns

In the final step of our studies, possibility of protein patterns formation by their selective adsorption to one of the blend components was verified. The analysis of protein adsorption to PMMA with different tacticities showed similar adsorption rate to at- and syn- PMMA, noticeably higher when compared with iso- PMMA. In turn, our previous publication shown that adsorption of proteins to PtBMA coatings only weakly depends on their stereoregularity and is very effective. Furthermore, protein adsorption to at-PtBMA is more efficient than to iso-PMMA films (Fig. S4). Therefore the blend of weakly adsorbing iso-PMMA and at-PtBMA capable of high protein adsorption, should enable formation of protein patterns.

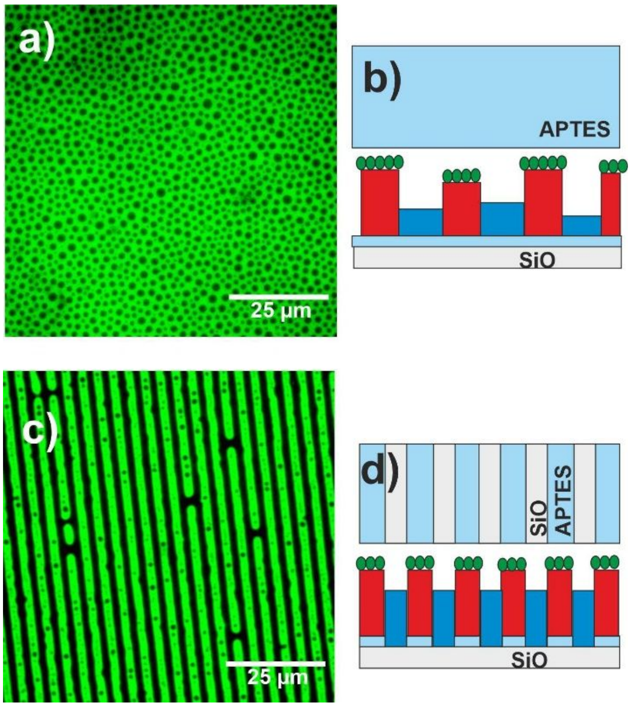

Figure 9. Protein adsorption to isotropic (a-b) and regular (c-d) phase domain patterns formed in iso-PMMA / at- PtBMA films h-dipped on homogeneous APTES (a-b) and patterns of alternating APTES/SiO stripes (c-d). Polymer domains with adsorbed protein traced using fluorescence microscopy (a, c) together with schematic illustration of the substrate and proteins adsorbed to the formed PtBMA domains (b, d).

This hypothesis was verified for both types of polymer patterns, i.e. isotropic and regular ones for two kinds of experiments. For regular patterns (stripes) the selective protein adsorption was verified by immersion of polymer film in fluorescence labeled rabbit IgG. After rising with PBS and DI water the sample was dried and fluorescence images were collected. In turn, for isotropic phase domains not only selective adsorption was verified but also biological activity of preabsorbed IgG was checked. This is the crucial and relevant issue for potential biomedical applications of protein patterns, since during adsorption protein molecules can change their orientation and as a result block access to specific part of a protein. To examine whether the

selective adsorption to prepatterned polymer surfaces reduces protein activity, the following experiment was performed. First, the iso-PMMA / at- PtBMA surface patterns were coated with rabbit anti-goat IgG and next incubated in a blocking buffer of BSA, which is commonly used to block any nonspecific interactions. Finally, the samples were incubated in solution of goat IgG (labeled with Alexa Fluor 488) dissolved in a blocking buffer. In such a procedure, fluorescently labeled goat IgG molecules bind only specifically with preabsorbed anti-IgG; therefore, the fluorescence images corresponds to position of preabsorbed rabbit anti-goat IgG and confirms their biological activity.

Recorded fluorescence micrographs (Fig. 9) show the patterns mirroring the phase domains observed on the surface and presented schematically in Fig. 9 b, d, thus confirming preferential adsorption of IgG molecules to PtBMA domains and confirmed their biological activity. This observation was evaluated qualitatively by comparison of one of Minkowski functionals, namely surface coverage F [63]. For this purpose original AFM topography images, Raman composition maps and adsorption fluorescence micrographs (upper row in Fig. 10) were transformed in the black-and-white images using the procedure described in our previous work [63], and then the fraction of white area was calculated, corresponding to elevated regions in AFM images, PtBMA domains in Raman composition maps and regions with adsorbed protein in fluorescence images. For both isotropic and regular pattern (Fig. 10) F showed the same values for all three types of images confirming the perfect match between topography and composition of observed domains and indicating perfect transposition of polymer pattern into protein array. These indicates preferential adsorption of proteins to PtBMA rich phase domains as well as retention of biological activity by adsorbed molecules, what is crucial step in fabrication of polymeric protein microarrays.

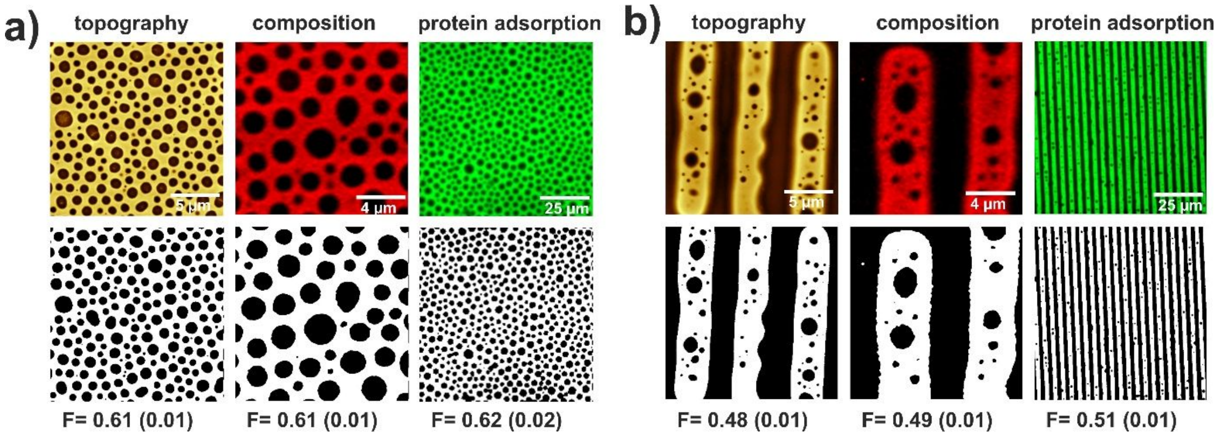

Figure. 10 Evaluation of quality of pattern transposition from isotropic (a) and regular (b) polymer patterns into protein arrays based on surface coverage analysis.

The described blend is an excellent example of the possibility of simplifying and improving the procedure for the production of polymeric protein microarrays by exploiting differences in the stereochemistry of the polymers. By using a suitable polymer mixture, in which the proteins adsorb preferentially to only one of the components (at-PtBMA), and by using a stamp of a given design, it is possible to obtain microarrays with high reproducibility.

CONCLUSIONS

In this work the systematic studies were performed, aimed at investigation of impact of PMMA tacticity on properties of thin PMMA films, their interactions with proteins and phase separation process in blends with PtBMA.

The thickness and topography of PMMA films, recorded using profilometer and AFM, respectively, revealed no differences between different tacticites. In turn, the surface chemistry of polymers films analyzed with ToF-SIMS combined with multivariate PCA analysis showed surface accumulation of polar ester functional groups for the at- and syn-PMMA films whereas these groups are hidden deeper for the iso-PMMA. These results were additionally confirmed by free surface energy calculations, which showed differences for the polar part of the SFE component, lower for iso-PMMA as compared to the at- and syn-PMMA films.

Then, the impact of tacticity on protein adsorption was studied for BSA, IgG and fibrinogen. The fluorescence micrographs revealed greater amount of protein molecules adsorbed to syn- and at-PMMA than to iso-PMMA films. In turn, ToF-SIMS analysis of protein orientation performed for BSA and IgG molecules showed different mechanisms of polymer-protein interactions caused by different protein structure and properties. The hydrophobic interactions were found to be responsible for the changes in BSA orientation whereas electrostatic interactions were more dominant in the orientation of IgG molecules. In the next step possibility of fabrication of protein microarrays based on polymer blends was examined using AFM and Raman microscopy, for blends composed of PMMA and PtBMA, both of different tacticities. Performed research showed that for SiO_x substrate phase separation process leads to formation bilayer structure for all PtBMA/PMMA blends, with lower PMMA layer covered with PtBMA. The 2-dimensional domains were observed only for at-PtBMA blended with PMMA with different tacticities when SiO_x was modified with APTES. This fact was used in the next step, where polymer blends were prepared on pre-patterned substrate with alternating SiO_x and APTES stripes. In this case, phase separation process driven by preferential interactions between PMMA and SiO_x led to formation of regular polymer patterns. The

elevated domains visible in topographical AFM images were composed of PtBMA, whereas the lowered regions were composed of PMMA as confirmed by Raman microscopy. Then, possibility of protein patterns formation by their selective adsorption to one of the blend components was verified showing perfect mirroring of both, isotropic and regular polymer patterns by proteins adsorbed to PtBMA domains. Finally, biological activity of adsorbed protein was confirmed, pointing to high potential of presented materials for application as platforms for protein microarrays.

CRediT authorship contribution statement

Natalia Janiszewska: Investigation, Visualization, Formal analysis, Methodology, Writing – original draft. **Joanna Raczkowska:** Writing – original draft, Formal analysis. **Katarzyna Gajos:** Investigation. **Kamil Awsiuk:** Conceptualization, Investigation, Formal analysis, Writing – original draft, Project administration, Funding acquisition, Supervision, Methodology.

Notes

The authors declare no competing financial interest.

ASSOCIATED CONTENT

Supporting Information

AFM images of PMMA films, ToF-SIMS signals selected for Principal Component Analysis, analysis of BSA denaturation, PCA loadings plot of positive ion ToF-SIMS spectra of whole antibody and their fragments, Fluorescence micrographs of IgG and BSA adsorbed onto iso- and at-PtBMA films, ToF-SIMS depth profiling of PtBMA:PMMA blends, images of PDMS stamp.

AUTHOR INFORMATION

Corresponding Author

Kamil Awsiuk - M. Smoluchowski Institute of Physics, Jagiellonian University, Łojasiewicza 11, 30-348 Kraków, Poland; Orcid http://orcid.org/0000-0001-9058-4561; Email: kamil.awsiuk@uj.edu.pl

Authors

Natalia Janiszewska - M. Smoluchowski Institute of Physics, Jagiellonian University, Łojasiewicza 11, 30-348 Kraków, Poland; Orcid http://orcid.org/0000-0001-5042-1848

Joanna Raczkowska - M. Smoluchowski Institute of Physics, Jagiellonian University, Łojasiewicza 11, 30-348 Kraków, Poland; Orcid http://orcid.org/0000-0002-2307-4614

Katarzyna Gajos - M. Smoluchowski Institute of Physics, Jagiellonian University, Łojasiewicza 11, 30-348 Kraków, Poland

Notes

The authors declare no competing financial interest.

ACKNOWLEDGMENTS

The study was carried out using research infrastructure purchased with the funds of the European Union in the framework of the Smart Growth Operational Programme, Measure 4.2; Grant No. POIR.04.02.00-00-D001/20, “ATOMIN 2.0 - ATOMic scale science for the INnovative economy”. This work was financially supported by the National Science Centre of Poland under Grants No. UMO-2016/21/D/ST5/01633.

REFERENCES

[1] E. Chiodi, A.M. Marn, M.T. Geib, M. Selim Ünlü, The role of surface chemistry in the efficacy of protein and dna microarrays for label-free detection: An overview, Polymers (Basel) 13 (2021) 1–21. https://doi.org/10.3390/polym13071026.

[2] J. Zemła, M. Lekka, J. Raczkowska, A. Bernasik, J. Rysz, A. Budkowski, Selective protein adsorption on polymer patterns formed by self-organization and soft lithography., Biomacromolecules 10 (2009) 2101–9. https://doi.org/10.1021/bm900598s.

[3] J.P. Gergen, R.H. Stern, P.C. Wensink, Filter replicas and permanent collections of recombinant DNA plasmids, Nucleic Acids Res 7 (1979) 2115–2136. https://doi.org/10.1093/nar/7.8.2115.

[4] H. Zhu, M. Snyder, Protein arrays and microarrays, Curr Opin Chem Biol 5 (2001) 40–45. https://doi.org/10.1016/S1367-5931(00)00170-8.

[5] J. LaBaer, N. Ramachandran, Protein microarrays as tools for functional proteomics, Curr Opin Chem Biol 9 (2005) 14–19. https://doi.org/10.1016/j.cbpa.2004.12.006.

[6] E. Chiodi, A.M. Marn, M.T. Geib, M. Selim Ünlü, The role of surface chemistry in the efficacy of protein and dna microarrays for label-free detection: An overview, Polymers (Basel) 13 (2021) 1–21. https://doi.org/10.3390/polym13071026.

[7] V. Romanov, S.N. Davidoff, A.R. Miles, D.W. Grainger, B.K. Gale, B.D. Brooks, A critical comparison of protein microarray fabrication technologies, Analyst 139 (2014) 1303–1326. https://doi.org/10.1039/c3an01577g.

[8] L. Filipponi, P. Livingston, O. Kašpar, V. Tokárová, D. V. Nicolau, Protein patterning by microcontact printing using pyramidal PDMS stamps, Biomed Microdevices 18 (2016) 1–7. https://doi.org/10.1007/s10544-016-0036-4.

[9] K.F.A. Clancy, S. Dery, V. Laforte, P. Shetty, D. Juncker, D. V Nicolau, Protein microarray spots are modulated by patterning method, surface chemistry and processing conditions, Biosens Bioelectron 130 (2019) 397–407. https://doi.org/https://doi.org/10.1016/j.bios.2018.09.027.

[10] K. Tsougeni, P.S. Petrou, K. Awsiuk, M.M. Marzec, N. Ioannidis, V. Petrouleas, A. Tserepi, S.E. Kakabakos, E. Gogolides, Direct Covalent Biomolecule Immobilization on Plasma-Nanotextured Chemically Stable Substrates, ACS Appl Mater Interfaces 7 (2015). https://doi.org/10.1021/acsami.5b01754.

[11] J. Zemła, J. Rysz, A. Budkowski, K. Awsiuk, Proteins grouped into a variety of regular micro-patterns by substrate-guided domains of self-assembling poly(ethylene oxide)/polystyrene blends, Soft Matter 8 (2012). https://doi.org/10.1039/c2sm25146a.

[12] K.F.A. Clancy, S. Dery, V. Laforte, P. Shetty, D. Juncker, D. V Nicolau, Protein microarray spots are modulated by patterning method, surface chemistry and processing conditions, Biosens Bioelectron 130 (2019) 397–407. https://doi.org/https://doi.org/10.1016/j.bios.2018.09.027.

[13] M. Rabe, D. Verdes, S. Seeger, Understanding protein adsorption phenomena at solid surfaces, Adv Colloid Interface Sci 162 (2011) 87–106. https://doi.org/10.1016/j.cis.2010.12.007.

[14] F. Rusmini, Z. Zhong, J. Feijen, Protein immobilization strategies for protein biochips, Biomacromolecules 8 (2007) 1775–1789. https://doi.org/10.1021/bm061197b.

[15] T.G. Tihan, M.D. Ionita, R.G. Popescu, D. Iordachescu, Effect of hydrophilic-hydrophobic balance on biocompatibility of poly(methyl methacrylate) (PMMA)-hydroxyapatite (HA) composites, Mater Chem Phys 118 (2009) 265–269. https://doi.org/10.1016/j.matchemphys.2009.03.019.

[16] R.Q. Frazer, R.T. Byron, P.B. Osborne, K.P. West, PMMA: An essential material in medicine and dentistry, J Long Term Eff Med Implants 15 (2005) 629–639. https://doi.org/10.1615/JLongTermEffMedImplants.v15.i6.60.

[17] M. Hassan, M. Asghar, S.U. Din, M.S. Zafar, Thermoset polymethacrylate-based materials for dental applications, in: Materials for Biomedical Engineering: Thermoset and Thermoplastic Polymers, Elsevier, 2019: pp. 273–308. https://doi.org/10.1016/B978-0-12-816874-5.00008-6.

[18] M. Unemori, Y. Matsuya, S. Matsuya, A. Akashi, A. Akamine, Water absorption of poly(methyl methacrylate) containing 4-methacryloxyethyl trimellitic anhydride, 2003.

[19] T. Kanie, H. Arikawa, K. Fujii, K. Inoue, Physical and mechanical properties of PMMA resins containing γ-methacryloxypropyltrimethoxysilane, J Oral Rehabil 31 (2004) 166–171. https://doi.org/10.1111/j.1365-2842.2004.01043.x.

[20] F. Pahlevanzadeh, H.R. Bakhsheshi-Rad, E. Hamzah, In-vitro biocompatibility, bioactivity, and mechanical strength of PMMA-PCL polymer containing fluorapatite and graphene oxide bone

cements, J Mech Behav Biomed Mater 82 (2018) 257–267. https://doi.org/10.1016/j.jmbbm.2018.03.016.

[21] M. Tavakoli, S.S.E. Bakhtiari, S. Karbasi, Incorporation of chitosan/graphene oxide nanocomposite in to the PMMA bone cement: Physical, mechanical and biological evaluation, Int J Biol Macromol 149 (2020) 783–793. https://doi.org/10.1016/j.ijbiomac.2020.01.300.

[22] S.M. Kenny, M. Buggy, Bone cements and fillers: A review, n.d.

[23] D.F. Farrar, J. Rose, Rheological properties of PMMA bone cements during curing, 2001.

[24] R.R. Trivedi, L. Werner, D.J. Apple, S.K. Pandey, Post cataract-and AM Izak 1 intraocular lens (IOL) surgery opacification, Eye 16 (2002) 217–241. https://doi.org/10.1038/sj/eye/6700066.

[25] P.M. Van Midwoud, A. Janse, M.T. Merema, G.M.M. Groothuis, E. Verpoorte, Comparison of biocompatibility and adsorption properties of different plastics for advanced microfluidic cell and tissue culture models, Anal Chem 84 (2012) 3938–3944. https://doi.org/10.1021/ac300771z.

[26] K. Tsougeni, P.S. Petrou, D.P. Papageorgiou, S.E. Kakabakos, A. Tserepi, E. Gogolides, Controlled protein adsorption on microfluidic channels with engineered roughness and wettability, Sens Actuators B Chem 161 (2012) 216–222. https://doi.org/10.1016/j.snb.2011.10.022.

[27] Z.K. Wang, H.Y. Zheng, H.M. Xia, Femtosecond laser-induced modification of surface wettability of PMMA for fluid separation in microchannels, Microfluid Nanofluidics 10 (2011) 225–229. https://doi.org/10.1007/s10404-010-0662-8.

[28] C. De Marco, R. Suriano, M. Levi, S. Turri, S. Eaton, G. Cerullo, R. Osellame, Femtosecond laser fabrication and characterization of microchannels and waveguides in methacrylate-based polymers, in: Microsystem Technologies, 2012: pp. 183–190. https://doi.org/10.1007/s00542-011-1347-2.

[29] M. Sivakumar, K. Panduranga Rao, In vitro release of ibuprofen and gentamicin from PMMA functional microspheres, J Biomater Sci Polym Ed 13 (2002) 111–126. https://doi.org/10.1163/156856202317414311.

[30] J. Sharma, X. Zhang, T. Sarker, X. Yan, L. Washburn, H. Qu, Z. Guo, A. Kucknoor, S. Wei, Biocompatible electrospun tactic poly(methyl methacrylate) blend fibers, Polymer (Guildf) 55 (2014) 3261–3269. https://doi.org/10.1016/j.polymer.2014.05.028.

[31] F. Fixe, M. Dufva, P. Telleman, C.B. Christensen, Functionalization of poly(methyl methacrylate) (PMMA) as a substrate for DNA microarrays., Nucleic Acids Res 32 (2004). https://doi.org/10.1093/nar/gng157.

[32] H. Matsuno, Y. Nagasaka, K. Kurita, T. Serizawa, Superior activities of enzymes physically immobilized on structurally regular poly(methyl methacrylate) surfaces, Chemistry of Materials 19 (2007) 2174–2179. https://doi.org/10.1021/cm0628956.

[33] T. Date, M. Ishikawa, K. Hori, K. Tanaka, T. Nagamura, M. Iwahashi, T. Serizawa, Water droplets for the symmetric adhesion of two poly(methyl methacrylate) films, Chem Lett 38 (2009) 660–661. https://doi.org/10.1246/cl.2009.660.

[34] T. Date, S. Yoshino, H. Matsuno, T. Serizawa, Surface modification of stereoregular and stereocomplex poly(methyl methacrylate) films with biologically identified peptides, Polym J 44 (2012) 366–369. https://doi.org/10.1038/pj.2011.147.

[35] T. Serizawa, T. Sawada, H. Matsuno, T. Matsubara, T. Sato, A peptide motif recognizing a polymer stereoregularity, J Am Chem Soc 127 (2005) 13780–13781. https://doi.org/10.1021/ja054402o.

[36] J.T. Peters, S.S. Hutchinson, N. Lizana, I. Verma, N.A. Peppas, Synthesis and Characterization of Poly(N-Isopropyl methacrylamide) Core/ Shell Nanogels for Controlled Release of Chemotherapeutics, Chemical Engineering Journal 340 (2018) 58–65. https://doi.org/10.1016/j.cej.2018.01.009.

[37] E.P. Ivanova, N. Mitik-Dineva, J. Wang, D.K. Pham, J.P. Wright, D. V. Nicolau, R.C. Mocanasu, R.J. Crawford, Staleya guttiformis attachment on poly(tert-butylmethacrylate) polymeric surfaces, Micron 39 (2008) 1197–1204. https://doi.org/10.1016/j.micron.2008.04.009.

[38] E.P. Ivanova, D.K. Pham, J.P. Wright, D. V. Nicolau, Detection of coccoid forms of Sulfitobacter mediterraneus using atomic force microscopy, FEMS Microbiol Lett 214 (2002) 177–181. https://doi.org/10.1016/S0378-1097(02)00878-9.

[39] D. V Nicolau, R. Cross, Protein profiled features patterned via confocal microscopy, 15 (2000) 85–91.

[40] N. Janiszewska, J. Raczkowska, K. Grzegorczyk, M. Brzychczy-Włoch, T. Gosiewski, M.M. Marzec, K. Gajos, K. Awsiuk, Effect of poly(tert-butyl methacrylate) stereoregularity on polymer film interactions with peptides, proteins, and bacteria, Colloids Surf B Biointerfaces 210 (2022) 112248. https://doi.org/10.1016/j.colsurfb.2021.112248.

[41] J. Zemła, M. Lekka, J. Wiltowska-Zuber, A. Budkowski, J. Rysz, J. Raczkowska, Integral geometry analysis of fluorescence micrographs for quantitative relative comparison of protein adsorption onto polymer surfaces, Langmuir 24 (2008) 10253–10258. https://doi.org/10.1021/la801313u.

[42] J. Rysz, M. Josiek, M.M. Marzec, E. Moons, Pattern replication in blends of semiconducting and insulating polymers casted by horizontal dipping, J Polym Sci B Polym Phys 51 (2013) 1419–1426. https://doi.org/10.1002/polb.23354.

[43] K. Awsiuk, P. Petrou, A. Thanassoulas, J. Raczkowska, Orientation of Biotin-Binding Sites in Streptavidin Adsorbed onto the Surface of Polythiophene Films, Langmuir 35 (2019). https://doi.org/10.1021/acs.langmuir.8b03509.

[44] K. Awsiuk, A. Budkowski, P. Petrou, M.M. Marzec, M. Biernat, T. Jaworska-Gołąb, J. Rysz, Orientation and biorecognition of immunoglobulin adsorbed on spin-cast poly(3-alkylthiophenes): Impact of polymer film crystallinity, Colloids Surf B Biointerfaces 148 (2016) 278–286. https://doi.org/10.1016/j.colsurfb.2016.08.028.

[45] D.K. Owens, R.C. Wendt, Estimation of the surface free energy of polymers, J Appl Polym Sci 13 (1969) 1741–1747. https://doi.org/10.1002/app.1969.070130815.

[46] X. Vanden Eynde, L.T. Weng, P. Bertrand, Infuence of Tacticity on Polymer Surfaces Studied by ToF-SIMS, Surface and Interface Analysis 25 (1997) 41–45.

[47] K. Yamashita, T. Serizawa, T. Kitayama, M. Akashi, Adsorption of Bovine Serum Albumin onto Poly (methyl methacrylate) Stereocomplex Films with a Molecularly, (2003) 1807–1812.

[48] H. Matsuno, Y. Nagasaka, K. Kurita, T. Serizawa, Superior activities of enzymes physically immobilized on structurally regular poly(methyl methacrylate) surfaces, Chemistry of Materials 19 (2007) 2174–2179. https://doi.org/10.1021/cm0628956.

[49] T. Serizawa, Y. Nagasaka, H. Matsuno, M. Shimoyama, K. Kurita, A stereocomplex platform efficiently detecting antigen-antibody interactions., Bioconjug Chem 18 (2007) 355–62. https://doi.org/10.1021/bc060225k.

[50] K. Awsiuk, A. Budkowski, M.M. Marzec, P. Petrou, J. Rysz, A. Bernasik, Effects of polythiophene surface structure on adsorption and conformation of bovine serum albumin: a multivariate and multitechnique study., Langmuir 30 (2014) 13925–33. https://doi.org/10.1021/la502646w.

[51] K. Gajos, K. Awsiuk, A. Budkowski, Controlling orientation, conformation, and biorecognition of proteins on silane monolayers, conjugate polymers, and thermo-responsive polymer brushes: investigations using TOF-SIMS and principal component analysis, Colloid Polym Sci 299 (2021) 385–405. https://doi.org/10.1007/s00396-020-04711-7.

[52] O.D. Monera, T.J. Sereda, N.E. Zhou, C.M. Kay, R.S. Hodges, Relationship of sidechain hydrophobicity and alpha-helical propensity on the stability of the single-stranded amphipathic alpha-helix., J Pept Sci 1 (1995) 319–329. http://www.scopus.com/inward/record.url?eid=2-s2.0-0029361842&partnerID=tZOtx3y1.

[53] K. Awsiuk, Y. Stetsyshyn, J. Raczkowska, O. Lishchynskyi, P. Dabczyński, A. Kostruba, H. Ohar, Y. Shymborska, S. Nastyshyn, A. Budkowski, Temperature-Controlled Orientation of Proteins on Temperature-Responsive Grafted Polymer Brushes: Poly(butyl methacrylate) vs Poly(butyl acrylate): Morphology, Wetting, and Protein Adsorption, Biomacromolecules 20 (2019). https://doi.org/10.1021/acs.biomac.9b00030.

[54] Protein Data Bank, (n.d.). www.rcsb.org/.

[55] E. Kosobrodova, R.T. Jones, A. Kondyurin, W. Chrzanowski, P.J. Pigram, D.R. McKenzie, M.M.M. Bilek, Orientation and conformation of anti-CD34 antibody immobilised on untreated and plasma treated polycarbonate., Acta Biomater 19 (2015) 128–37. https://doi.org/10.1016/j.actbio.2015.02.027.

[56] K. Gajos, K. Sanocka, M. Wytrwał, P. Dąbczyński, A. Budkowski, pH-dependent orientation of physisorbed and chemisorbed antibodies on silicon determined with TOF-SIMS and its effect on in-flow capture assay monitored with WLRS sensor, Appl Surf Sci 656 (2024). https://doi.org/10.1016/j.apsusc.2024.159644.

[57] K. Rezwan, L.P. Meier, M. Rezwan, J. Vörös, M. Textor, L.J. Gauckler, Bovine serum albumin adsorption onto colloidal Al 2O 3 particles: A new model based on zeta potential and UV-Vis measurements, Langmuir 20 (2004) 10055–10061. https://doi.org/10.1021/la048459k.

[58] E. Gasteiger, C. Hoogland, A. Gattiker, S. Duvaud, M.R. Wilkins, R.D. Appel, A. Bairoch, The Proteomics Protocols Handbook, Humana Press, 2005.

[59] M. Sprenger, S. Walheim, A. Budkowski, U. Steiner, Hierarchic Structure Formation in Binary and Ternary Polymer Blends, Interface Science 11 (2003) 225–235. https://doi.org/10.1023/A:1022182930454.

[60] S. Sugimoto, M. Inutsuka, D. Kawaguchi, K. Tanaka, Reorientation Kinetics of Local Conformation of Polyisoprene at Substrate Interface, ACS Macro Lett 7 (2018) 85–89. https://doi.org/10.1021/acsmacrolett.7b00927.

[61] P. Cyganik, A. Budkowski, U. Steiner, J. Rysz, A. Bernasik, S. Walheim, Z. Postawa, J. Raczkowska, J.R. Budkowski, U Steiner, J Rysz, A Bernasik, S Walheim, Z Postawa, Substructure formation during pattern transposition from substrate into polymer blend film, Europhysics Letters (EPL) 62 (2003) 855–861. https://doi.org/10.1209/epl/i2003-00451-1.

[62] J. Raczkowska, P. Cyganik, A. Budkowski, A. Bernasik, J. Rysz, I. Raptis, P. Czuba, K. Kowalski, Composition effects in polymer blends spin-cast on patterned substrates, Macromolecules 38 (2005) 8486–8493. https://doi.org/10.1021/ma051242s.

[63] J. Raczkowska, J. Rysz, A. Budkowski, J. Lekki, M. Lekka, A. Bernasik, K. Kowalski, P. Czuba, Surface Patterns in Solvent-Cast Polymer Blend Films Analyzed with an Integral-Geometry Approach, Macromolecules 36 (2003) 2419–2427. https://doi.org/10.1021/ma020870w.
