# Supplementary material for: Protein Orientation and Polymer Phase Separation Induced by Poly(methyl methacrylate) Tacticity
Source: Langmuir. 2025 Feb 3;41(5):3549–60. doi: 10.1021/acs.langmuir.4c04699 (PMC11823595; doi:10.1021/acs.langmuir.4c04699)
Supplement: Supplementary file 2 — la4c04699_si_002.pdf [file la4c04699_si_002.pdf]

SUPPORTING INFORMATION

Protein orientation and polymer phase separation induced by poly(methyl methacrylate) tacticity

Natalia Janiszewska[1,2], Joanna Raczkowska[1], Katarzyna Gajos[1], Kamil Awsiuk[1*]

[1]Jagiellonian University, Faculty of Physics, Astronomy and Applied Computer Science, Smoluchowski Institute of Physics, Łojasiewicza 11, 30-348 Kraków, Poland
[2]Jagiellonian University, Doctoral School of Exact and Natural Sciences, Łojasiewicza 11, 30-348
Kraków, Poland
[*] Corresponding author E-mail: kamil.awsiuk@uj.edu.pl

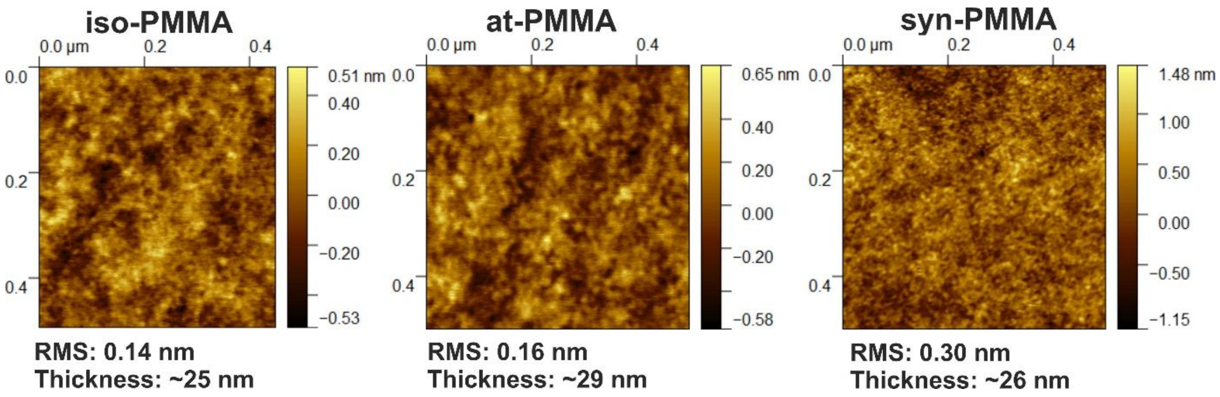

Figure S1. *Topographic AFM images of thin (a) at-. (b) iso- and (c) syn-PMMA films with measured thickness.*

Table S1. *ToF-SIMS signals and their mases selected for Principal Component Analysis from mass spectra of PMMA films.*

Ion	Mass (u)	Ion	Mass (u)
O^-	15.9955	C_4H^-	49.0095
OH^-	17.0033	$C_4H_2^-$	50.0156
$C_2H_3^-$	27.0253	$C_4H_3^-$	51.0254
CHO^-	29.004	$C_4H_4^-$	52.0313
CH_3O^-	31.0231	$C_4H_5^-$	53.042
O_2^-	31.9894	$C_3H_3O^-$	55.0233
C_3^-	36.0009	C_5^-	60.0022
C_3H^-	37.0088	C_5H^-	61.013
$C_3H_2^-$	38.0167	$C_5H_2^-$	62.017
$C_3H_3^-$	39.0254	$C_5H_3^-$	63.0248
C_2O^-	39.9956	$C_5H_4^-$	64.0318
CO_2^-	43.9915	$C_5H_5^-$	65.0416
C_4^-	48.0006	$C_5H_6^-$	66.0454

Table S2. *Main chain and ester group parameters for negative ToF-SIMS spectra of PMMA films and their ratios.*

	**iso-PMMA**	**at-PMMA**	**syn-PMMA**
**Chain**	0.495(0.009)	0.454(0.009)	0.451(0.009)
**Pendant group**	0.505(0.039)	0.546(0.041)	0.549(0.042)
**Pendant group/Chain ratio**	1.021(0.078)	1.201(0.065)	1.218(0.065)

Table S3. *ToF-SIMS signals, their mases and amino acid association selected for Principal Component Analysis from mass spectra of BSA adsorbed on PMMA films.*

**Ion**	**Mass (u)**	**Amino acid**
$C_3H_6N^+$	56.0488	Ala
$C_3H_4NO^+$	70.0264	Asn
$C_4H_8N^+$	70.069	Ile,Phe, Pro
$C_4H_{10}N^+$	72.0849	Val
$C_3H_8NO^+$	74.0592	Thr
$C_4H_5N_2^+$	81.046	His
$C_4H_6N_2^+$	82.052	His
$C_4H_6NO^+$	84.0416	Glu, Thr
$C_5H_{12}N^+$	86.1021	Ile,Leu
$C_3H_7N_2O^+$	87.0589	Asn
$C_3H_6NO_2^+$	88.0379	Asn, Asp
$C_4H_4NO_2^+$	98.0228	Asn
$C_6H_{12}N^+$	98.1002	Lys
$C_4H_{10}N_3^+$	100.089	Arg
$C_8H_{10}N^+$	120.0767	Phe
$C_9H_7O^+$	131.0496	Phe
$C_8H_{10}NO^+$	136.0721	Tyr

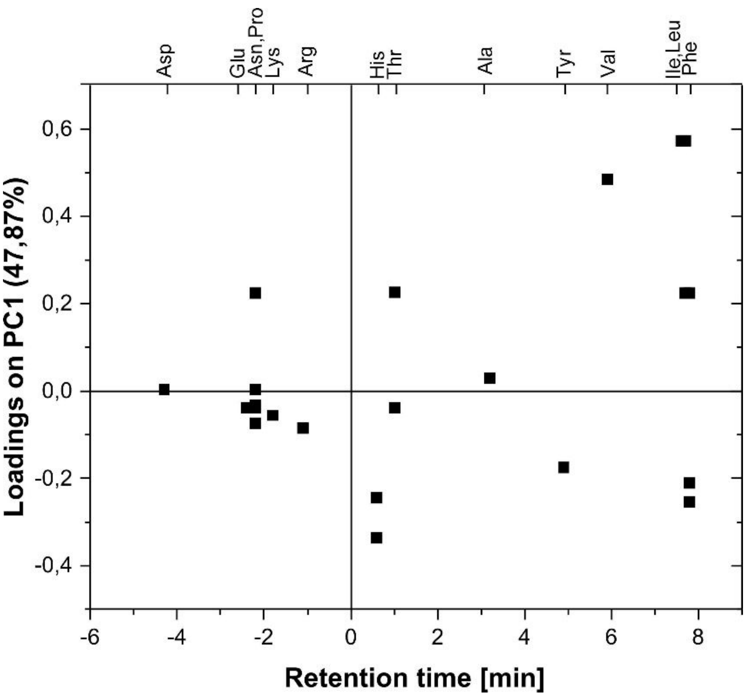

Figure S2. *Hydrophobicity of amino acid side chains defined as the difference in retention time (ΔtR) relative to glycine peptide plotted against the loadings on PC 1 for BSA adsorbed to PMMA thin films.*

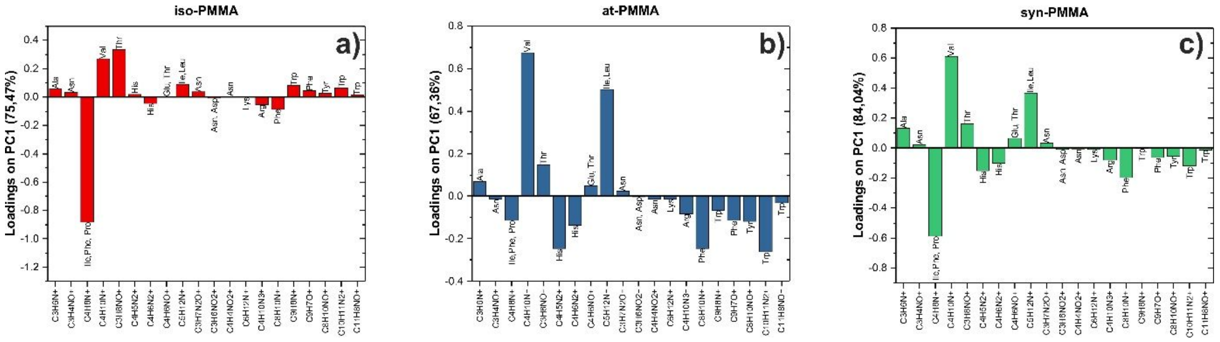

Figure S3. *PCA loadings plot of positive ion ToF-SIMS spectra of whole antibody, their Fab and Fc fragments adsorbed into iso-PMMA (a), at-PMMA (b) and syn-PMMA (c).*

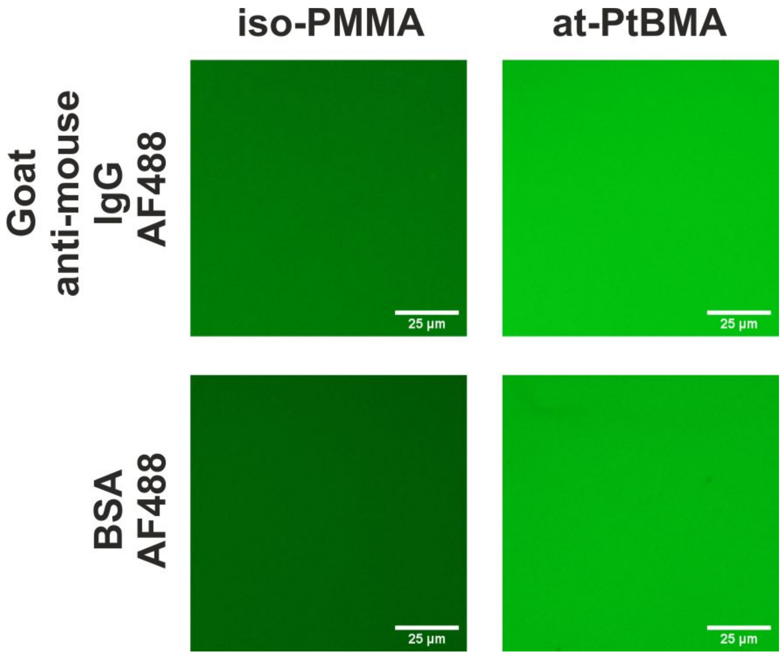

Figure S4. *Fluorescence micrographs show higher IgG and BSA adsorption to at-PtBMA than to iso-PMMA films.*

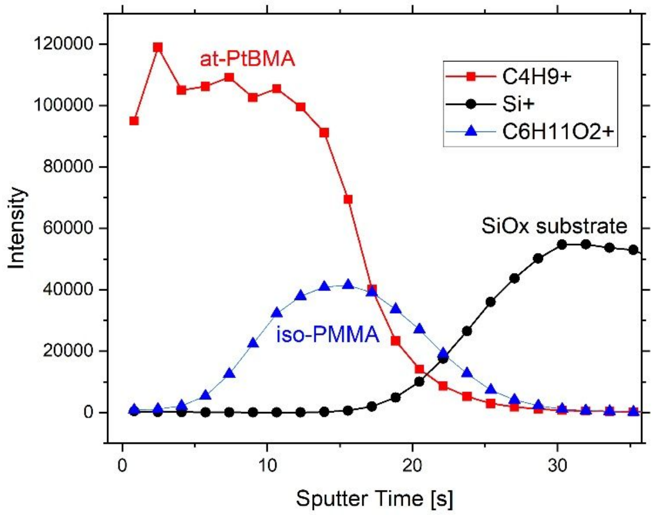

Figure S5. *ToF-SIMS depth profiling of PtBMA:PMMA blends on the SiOx substate.*

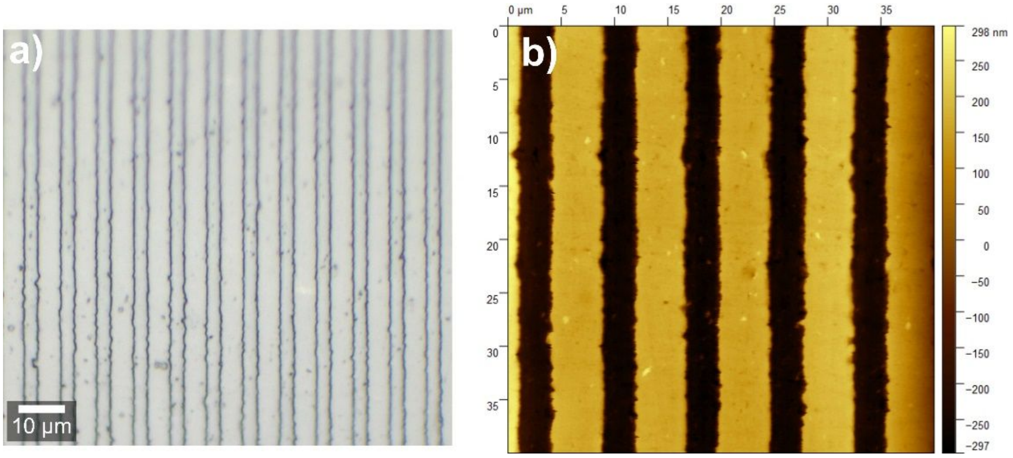

Figure S6. *Photograph (a) and AFM scan (b) of the asymmetric stamp with the stripe-like relief.*
